# Inflammatory dysregulation of blood monocytes in Parkinson’s disease patients

**DOI:** 10.1007/s00401-014-1345-4

**Published:** 2014-10-05

**Authors:** Veselin Grozdanov, Corinna Bliederhaeuser, Wolfgang P. Ruf, Valerie Roth, Kathrin Fundel-Clemens, Lisa Zondler, David Brenner, Ana Martin-Villalba, Bastian Hengerer, Jan Kassubek, Albert C. Ludolph, Jochen H. Weishaupt, Karin M. Danzer

**Affiliations:** 1 Department of Neurology, Ulm University, Albert Einstein Allee 11, 89081 Ulm, Germany; 2 Molecular Neurobiology Unit, German Cancer Research Center, 69120 Heidelberg, Germany; 3 Boehringer Ingelheim Pharma GmbH & Co. KG, Div. Research Germany, 88400 Biberach/Riss, Germany

**Keywords:** Parkinson’s disease, Blood monocytes, Inflammation, CCL2, FAS/FASLG

## Abstract

Despite extensive effort on studying inflammatory processes in the CNS of Parkinson’s disease (PD) patients, implications of peripheral monocytes are still poorly understood. Here, we set out to obtain a comprehensive picture of circulating myeloid cells in PD patients. We applied a human primary monocyte culture system and flow cytometry-based techniques to determine the state of monocytes from PD patients during disease. We found that the classical monocytes are enriched in the blood of PD patients along with an increase in the monocyte-recruiting chemoattractant protein CCL2. Moreover, we found that monocytes from PD patients display a pathological hyperactivity in response to LPS stimulation that correlates with disease severity. Inflammatory pre-conditioning was also reflected on the transcriptome in PD monocytes using next-generation sequencing. Further, we identified the CD95/CD95L as a key regulator for the PD-associated alteration of circulating monocytes. Pharmacological neutralization of CD95L reverses the dysregulation of monocytic subpopulations in favor of non-classical monocytes. Our results suggest that PD monocytes are in an inflammatory predisposition responding with hyperactivation to a “second hit”. These results provide the first direct evidence that circulating human peripheral blood monocytes are altered in terms of their function and composition in PD patients. This study provides insights into monocyte biology in PD and establishes a basis for future studies on peripheral inflammation.

**Electronic supplementary material:**

The online version of this article (doi:10.1007/s00401-014-1345-4) contains supplementary material, which is available to authorized users.

## Introduction

Parkinson’s disease (PD) is a chronic progressive neurodegenerative disease characterized by a progressive loss of neurons in the substantia nigra and other brain regions. However, the underlying pathomechanisms leading to neuronal death are currently unknown. While several mechanisms (e.g., protein aggregation, mitochondrial dysfunction and others) have been discussed as potential pathophysiological pathways, emerging evidence indicates that the immune system also plays a critical role in the pathogenesis of PD. For example, CNS neuroinflammation is recognized as a hallmark in PD [3] and genome-wide association studies have linked polymorphisms in immune genes as risk factors for PD [42]. In contrast to the wealth of studies on neuroinflammatory processes in the CNS of PD patients (see review [17]), the particular state of the peripheral innate immune system during PD is not understood. To date, some studies have shown elevated levels of serum cytokines (IL-2, IL-4, IL-6, IL-10, TNFα) in PD patients [11, 35, 40] as first general evidence of immune dysregulation. Specifically, peripheral blood mononuclear cells (PBMCs) are suspected to contribute to peripheral cytokine elevation but conclusive evidence in humans is largely missing. In an animal model for PD [18] and other neurodegenerative diseases [2, 9, 38], invasion of peripheral monocytes into the CNS has been demonstrated. Butovsky et al. [10] have clearly shown that monocytes which migrate into the mouse CNS are a distinct functional entity with different functional properties than CNS-resident microglia. Peripherally activated monocytes could thus have a tremendous impact on CNS inflammation but the existence and mode of peripheral innate inflammation in PD patients remain unclear to date.

In mice, two subsets of circulating monocytes based on their differential expression of CCR2 and CX3CR1 can be distinguished. The so-called “inflammatory monocytes” (Ly6C^hi^CCR2-^high^Cx3CR1^low^) with a pronounced pro-inflammatory cytokine profile are highly mobile and rapidly recruited to inflamed tissue. They can be differentiated from “resident” monocytes (Ly6C^lo^CCR2^low^CX3CR1^high^) that are important for regeneration and patrol along blood vessels [4, 24, 36]. Interestingly, recruitment of Ly6C^hi^ monocytes into the inflamed CNS has previously been reported in mouse models [10, 32]. In humans, equivalent subsets of monocytes can be distinguished based on the expression of CD14 and CD16. In detail, Ly6C^hi^ and LyC6^lo^ correspond to CD14+CD16− and CD14+CD16+ and are known as “classical” and “non-classical” monocytes, respectively [13].

Little is known about how different monocyte subpopulations are balanced in humans. The FAS/FAS ligand (FASLG) system can contribute to inflammatory reactions by mediating the release of cytokines from myeloid cells [34] and triggering the recruitment of peripheral monocytes and macrophages to the site of inflammation [5, 12]. Upregulation of FASLG expression in peripheral blood myeloid cells in rodents and in humans was found to be required for the recruitment of peripheral myeloid cells to the injured tissue. FASLG contributes to neuronal cell loss by regulating the differentiation and function of peripheral myeloid cells, rather than via direct FAS-induced apoptosis in FAS-bearing neural cells [31]. Even though there have been studies on the FAS/FASLG system in the context of apoptosis and PD in animal models [20, 28, 30], its involvement in inflammation and dysregulation in PD patients is still elusive. Specifically, the effect of FASLG on myeloid cells in the periphery and its potential role in the regulation of different monocyte subpopulations in humans have not yet been investigated.

Therefore, the aim of this study was a comprehensive characterization of circulating myeloid cells of PD patients and to understand their particular state during PD. Using a primary model for human monocytes, we examined the correlation between human monocyte functionality and clinical features of PD. We applied next-generation sequencing to explore the transcriptome of PD monocytes compared to age-matched healthy controls. We further studied the involvement of the FAS/FASLG system in monocyte regulation in PD and assessed its therapeutic consequences on monocyte function.

## Materials and methods

All chemicals used were purchased from Sigma Aldrich (Munich, Germany) unless stated otherwise.

### Ethical approval and study cohort

All human experiments were performed in accordance with the declaration of Helsinki and approved by the Ethics Committee of Ulm University. All study participants gave informed written consent to participate in the study.

PD patients as well as healthy controls were recruited at the University and rehab hospital Ulm (RKU). Healthy controls were chosen to match the patient cohort’s characteristics. A detailed description of the patient cohort for each experiment can be found as Supplementary Tables 1–5. In general, PD patients with confounding factors affecting the immune system were excluded for all experiments. PD patients with other co-morbidities like hypertension or depression were included in the study (see Supplementary Tables 1–5). All PD patients included in the study were diagnosed using the UK PD Society Brain Bank clinical diagnostic criteria at the RKU which is a specialized center for PD. Using a standard procedure peripheral venous blood in non-fasting state was collected with a Monovette™ blood drawing system (Sarstedt, Germany) and processed within 3 h post-collection. For isolation of peripheral blood leukocytes, EDTA was used as anticoagulant. Li-Heparin monovettes (Sarstedt, Germany) were used for the collection of whole blood for flow cytometry. For determination of peripheral blood levels of cytokines, serum collection tubes (Sarstedt, Germany) were used. Patients and healthy controls were age and sex matched. For age matching, both groups were designed to have no significant difference in age when comparing average, median and range. For sex matching, differences between investigated groups were no more than 10 % in advantage of either gender.

### Staging of PD progression with Hoehn & Yahr staging system

To assess the progression of PD in patients, the modified Hoehn & Yahr staging system was used [29]. A score of 0 indicates no detectable signs of disease and was assigned to all healthy controls. Disease progression is broken down to seven stages (including 1.5 and 2.5 in the modified version of the system) ranging from mild unilateral involvement (1.0) to confinement to wheelchair or bed (5.0).

### Isolation of peripheral blood mononuclear cells and purification of peripheral blood monocytes

Peripheral blood mononuclear cells were isolated from venous whole blood by density centrifugation on Histopaque^®^ density gradient. After two subsequent washes to get rid of contaminating platelets, cells were labeled with magnetic beads coupled to monoclonal antibodies against CD14 (Miltenyi Biotec, Germany) and CD14-positive cells separated in a column placed in a strong magnetic field (Miltenyi Biotec, Germany). Subsequently, the column was removed from the magnetic field and the retracted cells eluted with MACS™ buffer (Miltenyi Biotec, Germany). For untouched monocytes isolation, non-monocytes were labeled with biotin-conjugated antibodies against CD3, CD7, CD16, CD19, CD56, CD123 and Glycophorin A, and Anti-Biotin MicroBeads (Miltenyi Biotec, Germany). The flow-through fraction represents the unlabeled monocytes. Cells were then centrifuged at 300 g for 10 min at RT and resuspended in cell culture medium (RPMI 1640, 10 % FCS (PAA, Austria), 1 % penicillin/streptomycin (PAA, Austria) or RLT-lysis buffer (Qiagen, The Netherlands) for further applications. The purity of the isolated cell population was controlled by flow cytometry analysis and immunocytochemistry.

### Monocyte culture and stimulation

To functionally characterize monocyte activation, monocytes were plated on uncoated culture plates (24-well, BD Biosciences, USA) at a density of 10^6^ cells/ml (5.10^5^ cells per 24-well). Monocytes were allowed to rest for 24 h prior to the LPS stimulation (1 ng/ml, [15]) to eradicate the influence of mechanical/chemical/temperature stress during the isolation procedure on experimental results. Monocytes were cultured in sterile RPMI 1640 medium supplemented with 10 % v/v fetal calf serum (FCS, PAA, Austria) and 1 % v/v penicillin–streptomycin (P/S; 1,000 U/ml, PAA, Austria). Sterile culture medium was used as a negative control for monocyte stimulation. LPS stimulation was performed in duplicates. After 24 h stimulation, the supernatant was collected and the cells were lysed with extraction lysis buffer (1 M NaCl, 0.6 % w/v sodium deoxycholate, 0.6 % v/v Igepal, 5 % v/v 1 M Tris pH 8.0, protease inhibitor cocktail (Roche, Switzerland)).

### Cytokine quantification in cell culture supernatants

Cytokine levels in culture supernatants were quantified by ELISA (IL-6, IL-10, CCL2, IFNγ, IL-1β; all antibody pairs obtained from BioLegend, USA) or with an electrochemiluminescence immunoassay (TNFα, IFN-8; MesoScale Discovery, USA). Each standard and sample were measured by means of two replicates, average value calculated and blank absorption of matrix subtracted. A threshold of 0.2 coefficient of variance (CV) was set as a limit to consider a value significant. Absorption values of known standards were plotted against concentrations and the resulting sigmoidal curve fitted using non-weighted five-parameter logistics with ReaderFit™ (Hitachi Solutions, Japan). Cytokine release was normalized to cell number by quantification of protein concentration in cell lysate, measured by BCA assay (Thermo Scientific, USA) according to the supplier’s instructions.

### FACS

Four milliliters of anticoagulated whole blood (Sarstedt, Germany) was collected in Li-Heparin monovettes from healthy volunteers and PD patients after informed consent. Osmotic lysis of red blood cells was performed with a red blood cell lysis buffer (RBC-LB: 50 mM NH_4_Cl, 70 mM NaHCO_3_, 0.1 % EDTA, dH_2_O). After lysis and washing with FACS buffer [PBS (Life Technologies) + 10 % FCS (PAA, Austria)], cells were resuspended in RPMI + 10 % FCS + 2 % P/S and treated 24 h or 48 h at 37 °C with vehicle control (PBS) or APG101 (250 µg/ml). After incubation cells were resuspended, washed with FACS buffer and stained with anti-human antibodies specific for anti-HLA-DR (1:200; Biolegend, USA, L243, Pe-Cy7), anti-CD14 (1:200; BD, USA; M5E2, PerCP-Cy5.5), anti-CD16 (1:10; BD, 3G8, PE), anti-CD2 (1:20; eBioscience, USA RPA-2.10, FITC), anti-CD335 (1:100; AbDSerotec, UK; 9E2, FITC), anti-CD15 (1:13; Miltenyi Biotec, VIMC6, FITC) and anti-CD19 (1:100; eBioscience, USA; HIB19, FITC) for 25 min at 4 °C in the dark. After washing, the stained cells were fixed 20 min in 2 % PFA, filtered (70 μm) and analyzed by flow cytometry (LSRII, BD) using appropriate color compensation to correct for spectral overlap and autofluorescence. Therefore, following isotype controls were used at the same concentration as the primary antibodies: mouse IgG_2a_, κ (Biolegend, USA MOPC-173, PE-Cy7), mouse IgG_1_, κ (eBioscience, USA, P3.6.2.8.1, FITC), mouse IgG_2a_, κ (BD, USA; G155-178, PerCP-Cy5.5), mouse IgG_1_, κ (BD, MOPC-21, PE). Monocytes are among HLA-DR+ cells that do not express B-cell (CD19−), T-cell (CD2−), NK-cell (CD335−), or granulocyte makers (CD15−). Within this gate, CD14+ CD16− and CD14+ CD16+  monocytes, CD14− CD16+ monocytes and CD14− CD16− DCs can be separated. To exclude cell doublets, cells were gated using area vs. width signal intensity. Data are presented as mean ± SEM from four independent experiments.

### Next-generation sequencing and expression analysis

For whole-transcriptome analysis of expression, monocytes were isolated from the peripheral blood of healthy controls and PD patients as described above and lysed with RLT buffer (Qiagen, The Netherlands). After isolation of total RNA (miRNeasy Mini Kit, Qiagen, The Netherlands), 200 ng total RNA was used for the generation of mRNA-focused libraries (TruSeq RNA Sample Preparation Kit v2, Set B, Illumina, USA). Clusters were generated with cBot (Illumina, USA) with the TruSeq SR Cluster Kit v3-cBot-HS (Illumina, USA). Samples were sequenced on a HiSeq 2000 sequencing system (Illumina, USA) with TruSeq SBS Kit HS-v3 (50 cycles). 52 bases were sequenced with single read and 2.0–2.5 × 107 reads per sample, 7 bases IndexRead.

For the unsupervised hierarchical clustering of subjects based on gene expression, genes with average expression among all samples <5 FPKM were excluded. Cosine correlation and complete linkage were used for clustering with Genesis [39]. Heatmaps and cluster diagrams were generated with Genesis. For pathway analysis, genes were filtered by expression level (≥5 in at least one of the two groups to be compared), log ratio (log2 ≥ 1 or ≤−1) and significance of the difference (*q* value, significance corrected for multiple testing by Benjamini Hochberg, *q* ≤ 0.05) between the compared groups. Canonical pathways and upstream regulators were predicted by an Ingenuity pathway analysis (Qiagen, The Netherlands); involved molecular functions and biological processes were identified with Genomatix genome analyser pathway system (Genomatix Software GmbH, Germany). For the identification of genes which are consistently expressed within the two groups to compare, we filtered out all genes whose expression pattern was determined by less than 25 % of the expression values. For heatmap generation, *z* scores were built for each value.

### RT-qPCR

Total RNA was extracted using RNeasy Mini Kit (Qiagen, USA) in accordance with the manufacturer’s instructions. RNA concentration and quality were controlled using NanoDrop (ThermoScientific, Waltham, MA, USA) and 1 µg of total RNA was used to synthesize cDNA using Iscript reverse transcriptase (Bio-Rad Laboratories) containing oligo-dT primers and random primers. cDNA was then diluted ten times and PCR analysis was performed on a Bio-Rad iCycler System using iQSYBR Green Supermix. A specific standard curve was performed in parallel for each gene to assess the specificity of the products, for quantification of the respective transcripts in duplicate. PCR conditions were 3 min at 95 °C, followed by 40 cycles of 30 s at 94 °C and 10 s at 60 °C (for FASLG, 50 cycles of 20 s). The relative levels of each gene of interest were normalized to TBP, GAPDH (Fig. 5) and B2M (Fig. 2). Primers for FAS, FASLG and GAPDH were obtained from Qiagen. A list of all other primers used is provided in Supplementary Table 8.

### Phagocytosis assay

Phagocytosis assay was performed as per supplier’s instructions with pHrodo™ Green Zymosan A BioParticles^®^ (Life Technologies, Carlsbad, USA) conjugate for phagocytosis. In brief, monocytes were plated on a 96-well tissue culture plate and incubated in RPMI1640 10 % FCS 1 %P/S containing fluorescently labeled zymosan particles (0.5 mg/ml) for 4 h at 37 °C, 5 % CO_2_. Fluorescence was measured with Victor X3™ microplate reader (Perkin-Elmer, Waltham, USA).

### Statistical analysis

Statistical analyses were carried out using GraphPad Prism version 6.02 for Windows, GraphPad Software, San Diego, CA, USA, “www.graphpad.com”. All values in the figures are presented as mean ± SEM unless stated otherwise. Outliers were identified by ROUT analysis with *Q* = 1 %. Gaussian distribution was tested with D’Agostino and Pearson omnibus normality test, Shapiro–Wilk normality test and KS normality test. Variances were compared with *F* test. Unpaired *t* test with (unequal SD) or without (equal SD) Welch’s correction was used for unpaired data and paired *t* test for paired data from Gaussian distribution. Mann–Whitney test (unpaired data) or Wilcoxon matched-pairs signed-rank test (paired data) was used for data from non-Gaussian distribution. All tests for significance were two tailed.

## Results

### Enrichment of classical CD14+CD16− monocytes in peripheral blood of PD patients

**Fig. 1:**
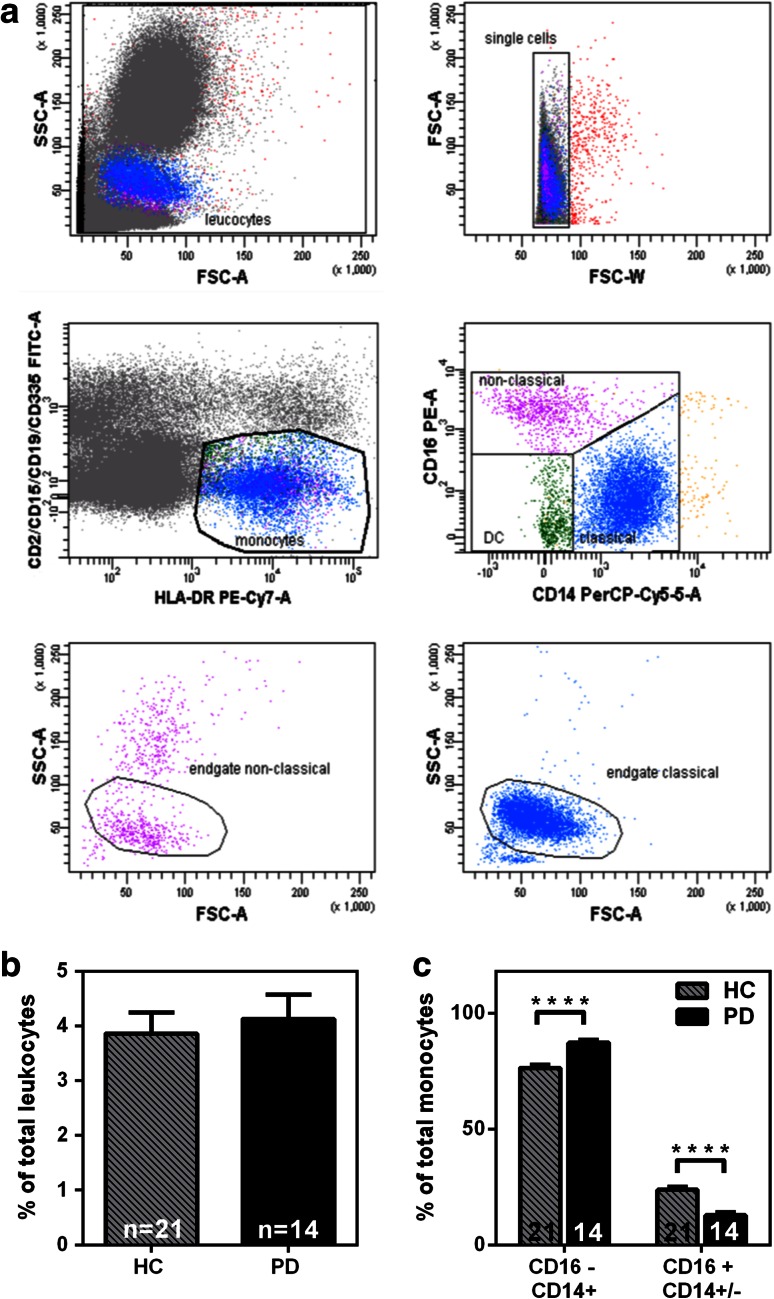
Classical (CD14+CD16−) monocytes are enriched in PD patients. **a** Representative FACS analysis of monocyte subpopulations from peripheral blood. Monocytes are gated out of other immune cells (FITC—CD2/CD15/CD19/CD335) by HLA-DR staining (PE-Cy7) and monocyte subpopulations characterized based on CD14 (PerCP-Cy5.5) and CD16 (PE) staining. **b** Total number of monocytes in peripheral blood as a percentage of total leukocytes. No significant difference between PD patients’ and healthy controls’ blood was found (Healthy controls *n* = 21, PD *n* = 14). **c** CD14+CD16− (classical) monocytes are enriched and CD14−/+CD16+ monocytes are decreased in the blood of PD patients. (Healthy controls *n* = 21, PD patients *n* = 14). *Bars* mean ± SEM, ****p < 0.0001

To investigate whether the composition of peripheral myeloid cells is altered in PD, we used a FACS-based analysis to characterize different monocyte subpopulations from PD patients and age-matched healthy controls. To avoid confounding factors affecting the immune system all PD patients with acute inflammatory co-morbidities were excluded from all following experiments. A detailed description of the PD patient cohort for each of the following experiments can be found as Supplementary Tables 1–5. We applied a FACS analysis strategy that was published recently [13], in which blood monocytes can be discriminated from the rest of the leukocytes based on high expression of HLA-DR and absence of B-cell (CD19−), T-cell (CD2−), NK-cell (CD335−), or granulocyte markers (CD15−). Within this cell population, classical (CD14+CD16−) and non-classical (CD14−CD16+) monocytes can be distinguished (Fig. 1a). We included the intermediate population (CD14+CD16+) to the non-classical population and refer to this combinatory population as CD16+ population according to Ziegler and Heitbrock [44]. Comparing total numbers of monocytes from PD patients and healthy controls revealed no significant difference (Fig. 1b). Interestingly, we found that in the peripheral blood of PD patients, classical monocytes (CD16−) are enriched, and concurrently, CD16+ monocytes are reduced compared to the monocyte composition from healthy donors (Fig. 1c). It has been suggested that a non-coding variant of HLA-DR A influences the expression of HLA-DR [1, 42]. To control that HLA-DR expression did not influence our FACS results, we quantified HLA-DR-positive monocytes in PD patients and healthy controls. As demonstrated in Supplementary Fig. 1, no significant difference in HLA-DR expression between the PD and control group was detected.

### PD monocytes exhibit a dysregulation of inflammatory pathways

**Fig. 2:**
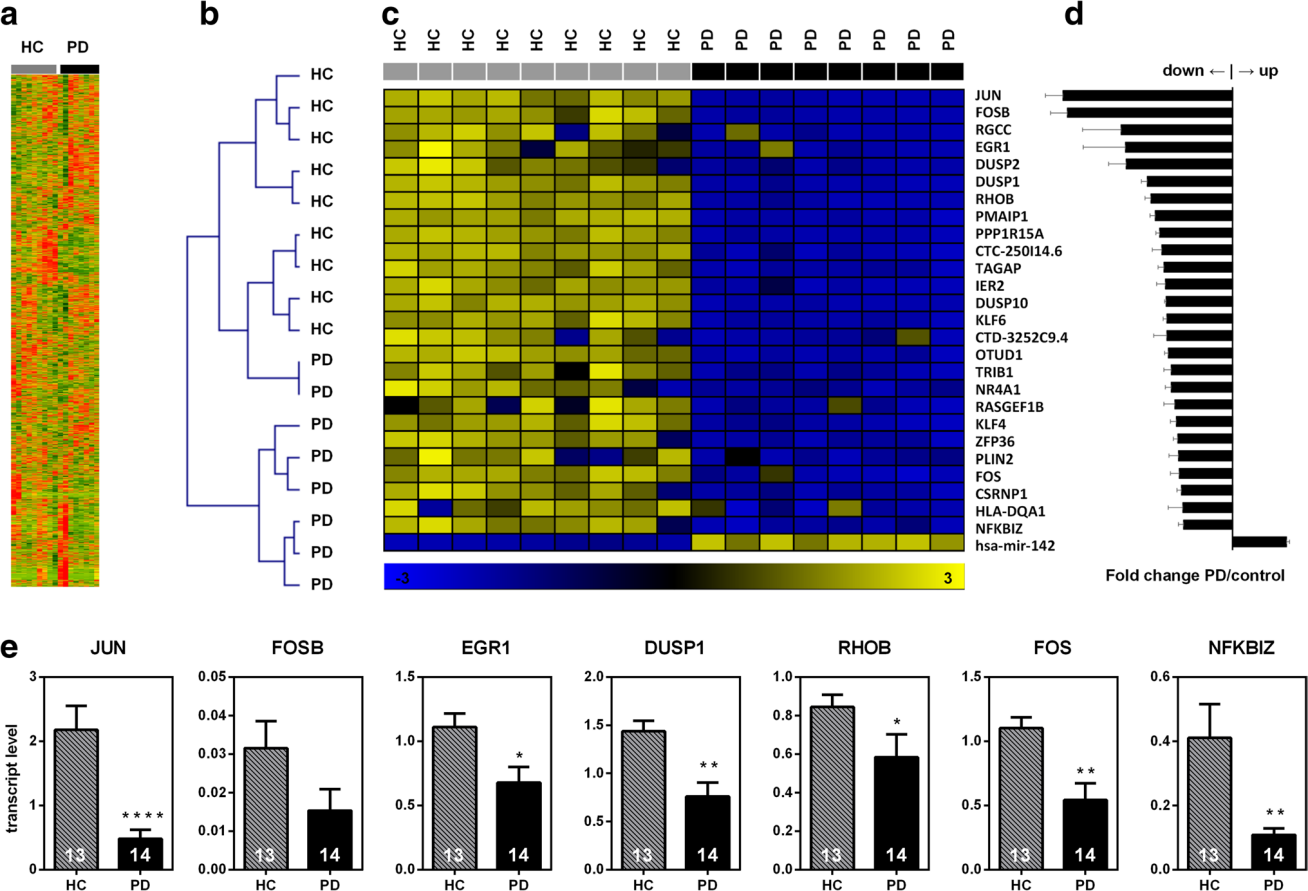
Transcriptome-wide analysis of gene expression in PD and healthy control monocytes by next-generation sequencing reveals a distinct expression signature in PD monocytes. **a** Heatmap of 8,862 genes expressed over the threshold (FPKM >5). **b** Unsupervised hierarchical clustering based on the expression of 8,862 genes in blood monocytes successfully identified most PD cases and clustered them away from healthy controls. Clustering by complete linkage and cosine correlation. **c** Differential expression of selected top genes in PD monocytes. **d** Fold change of expression in PD monocytes vs. healthy controls’ monocytes. **e** Validation of selected genes’ expression in an independent cohort (healthy controls *n* = 13, PD patients *n* = 14) by qRT-PCR. *Bars* represent mean ± SEM, *p < 0.05, **p < 0.01, ****p < 0.0001

To investigate the transcriptome profile of peripheral monocytes in PD and to identify molecular pathways which are differentially regulated compared to healthy controls, we purified peripheral blood monocytes from PD patients (*n* = 8) and healthy controls (*n* = 9) by positive magnetic selection, isolated total RNA and subjected it to next-generation sequencing. Detected transcripts were mapped to a whole-genome library and a total of 8,862 genes were expressed over the lower threshold (average FPKM >5, Fig. 2a). Strikingly, unsupervised hierarchical clustering (complete linkage by cosine correlation) revealed similar expression patterns among PD patients and largely separated disease cases from healthy controls with only two exceptions (Fig. 2b).

A detailed analysis based on filtering genes by expression and significant difference between the disease and control groups revealed that genes which are differentially expressed in PD monocytes include transcription factors, secreted proteins and micro-RNAs (Fig. 2c, d).

Pathway (Table 1) and upstream (Table 2) analysis (IPA) demonstrated that the most significantly affected pathways are related to inflammation: granulocyte and agranulocyte adhesion, diapedesis, regulation of cytokine production, response to chemical stimulus and organic substances, response to stress and to LPS (Supplementary Table 6). Bacterial LPS, TGFβ, TNFα, PDGF and FAS were identified as most probably involved signal mediators (Table 2). In addition, we found that chemokine binding and activity, MAP-kinase signaling and cytokine signaling are dysregulated in PD monocytes (Supplementary Table 7).

**Table 1:** Top 5 differentially regulated canonical pathways in PD monocytes (ingenuity pathway analysis)

Ingenuity canonical pathways	*p* value of overlap
Granulocyte adhesion and diapedesis	5.89E−09
Agranulocyte adhesion and diapedesis	1.05E−08
Differential regulation of cytokine production in macrophages and T helper cells by IL-17A and IL-17F	1.35E−08
Differential regulation of cytokine production in intestinal epithelial cells by IL-17A and IL-17F	5.25E−08
IL-17A signaling in fibroblasts	4.79E−07

**Table 2:** Top 5 predicted upstream regulators in PD monocytes (ingenuity upstream analysis)

Upstream regulator	*p* value of overlap	Target molecules in dataset
Lipopolysaccharide	3.57E−37	ATF3, CCL2, CCL3, CCL4, CD83, CD9, COL1A2, COL6A1, CTSK, CXCL1, CXCL2, DCN, DKK3, DUSP1, DUSP2, EGR1, EGR2, FOS, FOSB, G0S2, GPR183, HCAR3, HLA-DQA1, IGFBP4, IL1B, IL8, JUN, KLF4, KLF6, MME, MMP2, MMP9, NFKBIA, NFKBIZ, NR4A1, NR4A2, OLR1, PLIN2, PMAIP1, PPP1R15A, PTGS2, RGS1, RHOB, SERPINH1, SLAMF7, SPARC, TGM2, TNC, TPM2, TRIB1, ZFP36
PDGF BB	3.22E−36	ATF3, CCL2, CXCL2, DCN, DUSP1, EGR1, EGR2, FHL1, FOS, FOSB, GPR183, IER2, IGFBP4, IL1B, IL8, JUN, KLF6, MMP2, MMP9, NR4A1, NR4A2, OLR1, PPP1R15A, PTGS2, RGS1, RHOB, SCD, TGM2, TNC, TRIB1, ZFP36
TGFB1	6.24E−31	CCL2, CCL3, CCL4, CD83, CLU, COL1A2, COL6A1, COL6A2, CTSK, CXCL1, CXCL2, DCN, DKK3, DUSP1, ECM1, EGR1, EGR2, EMILIN1, FHL1, FOS, FOSB, HLA-DQA1, IER2, IGFBP4, IL1B, IL8, JUN, KLF4, LTBP2, MMP2, MMP9, NFKBIA, NR4A1, NR4A2, OLR1, PTGS2, RGCC, RHOB, SCD, SERPINH1, SERTAD1, SPARC, TGM2, TNC, TPM2, ZFP36
LDL	4.04E−30	ATF3, CCL2, CCL3, CCL4, CTSK, CXCL2, DUSP1, DUSP2, EGR1, FOS, FOSB, G0S2, GPR183, IGFBP4, IL1B, IL8, JUN, MMP2, MMP9, NR4A1, NR4A2, OLR1, PLIN2, PMAIP1, PTGS2
FAS	1.10E−29	CCL2, CCL3, CCL4, CD83, COL1A2, COL6A1, CXCL1, CXCL2, DUSP10, EGR1, EGR2, FOS, FOSB, GPNMB, HCAR3, IL1B, IL8, JUN, MME, MMP9, NFKBIA, NR4A1, NR4A2, PLIN2, PPP1R15A, TNC, TRIB1, ZFP36

In accordance with the next-generation sequencing results from the PD discovery cohort, we could confirm by qRT-PCR in a separate validation cohort of 14 PD patients and 13 controls the highly significant downregulation of six mRNAs: JUN, EGR1, DUSP1, RHOB, FOS, NFKBIZ and a tendency for downregulation for FOSB (Fig. 2e).

### PD monocytes are pathologically hyperactive in response to LPS stimulation and their hyperactivity is correlated to PD severity

**Fig. 3:**
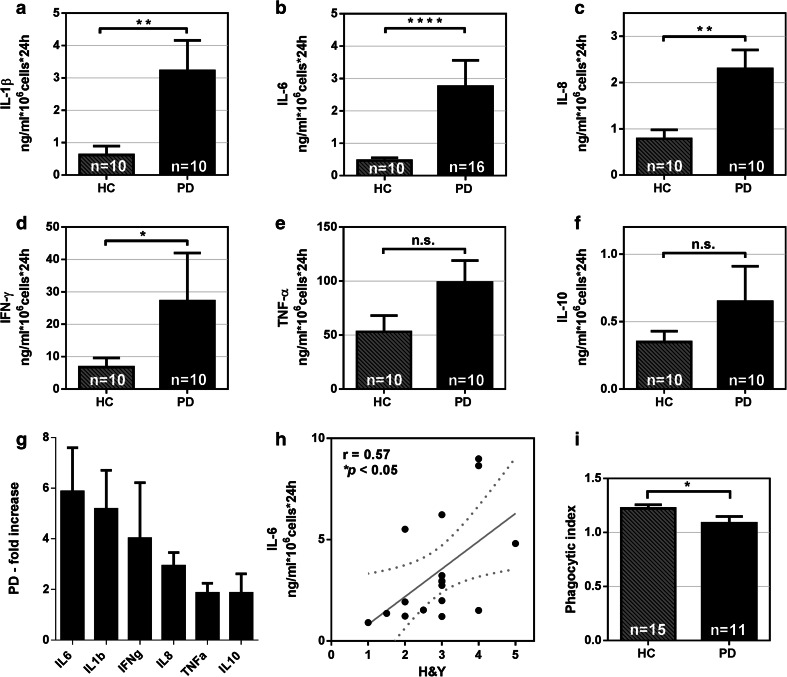
PD monocytes are hyperactive upon LPS stimulation. Monocytes from PD patients secrete significantly higher levels of IL-1β, IL-6, IL-8 and IFNγ when stimulated with 1 ng/ml LPS compared to monocytes from age-matched healthy controls (**a–d**). No significant difference in cytokine secretion was observed for TNFα (*p* = 0.05), and IL-10 (*p* = 0.52) (**e, f**). *Bars* represent mean ± SEM. PD patients (*n* = 10), healthy controls (*n* = 10), *p < 0.05, **p < 0.01, *** *p* < 0.001, ****p < 0.0001. **g** Fold increase in cytokine production of monocytes from PD patients vs. healthy controls. **h** Correlation between IL-6 release after 24 h stimulation with 1 ng/ml LPS and PD severity staged with the H&Y scale. Dotted lines indicate the 95 % CI of the linear regression. *r* Spearman’s correlation coefficient. **i** Decreased phagocytic function of Parkinson’s disease monocytes, healthy controls (*n* = 15), PD patients (*n* = 11). *Bars* represent mean ± SEM, *p < 0.05

To explore the physiological relevance of our findings and to test the hypothesis that the function of myeloid cells is altered in PD patients, we established a human primary monocyte culture system using positive magnetic selection based on CD14 expression and subsequent culture. The purity of the monocytic fraction was higher than 95 % (Supplementary Fig. 2a–c) and all monocytic subpopulations were present in the monocytic fraction even when CD14 is expressed at low levels in non-classical monocytes (Supplementary Fig. 2d). To exclude the possibility that anti-CD14 magnetic beads impair monocyte activation or pre-activate monocytes, IL-6 release was measured after LPS stimulation using both anti-CD14 positive and negative isolation methods. No difference in monocyte activation was detected, thereby ensuring the quality of our assay system (Supplementary Fig. 2e). Monocytes from PD patients and controls were then stimulated with LPS in culture. Strikingly, we found that monocytes from PD patients behave abnormally, displaying excessive pro-inflammatory IL-1β, IL-6, IL-8 and IFNγ production compared to monocytes from age- and sex-matched healthy controls (Fig. 3a–d). Even though statistical significance was not reached (*p* = 0.06), a trend for increased TNFα and IL-10 secretion was also observed for PD monocytes compared to control monocytes (Fig. 3e–f). The most striking increase of cytokine release upon LPS stimulation from PD monocytes was found regarding IL-6 production (*p* < 0.0001) (Fig. 3g). Since most PD patients included in the study were on l-dopa treatment (see Supplementary Tables 1–5), we also tested the effect of l-dopa on IL-6 release in our monocyte cultures to rule out the possibility that l-dopa leads to an excess release of IL-6. As shown in Supplementary Fig. 3, l-dopa had no significant effect on IL-6 release of monocytes from healthy donors. Interestingly, we did not detect any excess production of cytokines by PD monocytes compared to monocytes from healthy controls without LPS stimulation.

We hypothesized that if monocyte hyperactivation upon LPS challenge is pathophysiologically relevant, the extent of monocyte hyperactivation should be reflected in clinical parameters. Indeed, we found that IL-6 secretion from LPS-stimulated monocytes derived from PD patients was strongly positively correlated with the Hoehn & Yahr (H&Y) stage of disease severity (Fig. 3h).

To further characterize the functional abnormalities of isolated PD monocytes, we assessed another main function of myeloid phagocytes—the phagocytosis of extracellular material. To this end, we applied a highly specific phagocytosis assay employing pH-sensitive-labeled yeast wall particles exhibiting a weak fluorescence at neutral pH and a dramatic increase in fluorescence at lower pH. This enables the quantification of cellular uptake and acidification of the phagocytic compartments. FACS analysis and microscopic pictures at the cellular level confirm the uptake of fluorescent particles (Supplementary Fig. 4). Using this phagocytosis assay, we found a significant reduction in phagocytic activity in the monocytes of PD patients compared to those of healthy controls (Fig. 3i).

### The CCR2–CCL2 axis is activated in PD

**Fig. 4:**
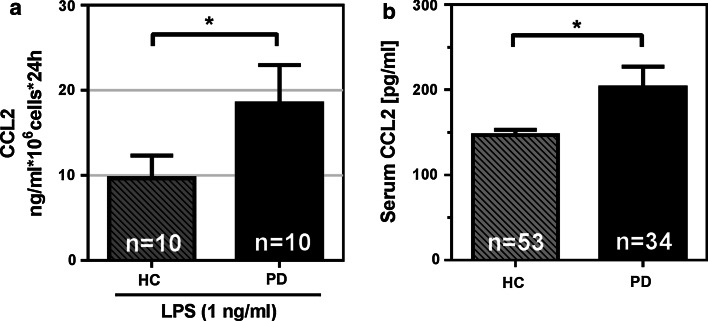
The CCR2–CCL2 axis is activated in PD. **a** Monocytes from PD patients release significantly higher levels of CCL2 upon LPS stimulation (LPS 1 ng/ml, healthy controls *n* = 10, Parkinson’s disease patients *n* = 10, *p < 0.05). **b** CCL2 is significantly increased (*p < 0.05) in the serum of PD patients (*n* = 53) when compared to healthy controls (*n* = 34). *Bars* represent mean ± SEM

Classical monocytes are characterized by high expression of C–C chemokine receptor type 2 (CCR2) and are recruited from the bone marrow in a chemokine-dependent manner. C–C motif ligand 2 (CCL2, also known as monocyte chemoattractant protein-1 MCP-1) is involved in monocyte recruitment from the bone marrow and spleen to the site of inflammation and is dependent on the presence of its receptor C–C chemokine receptor type 2 (CCR2) [43]. To further support the idea that PD patients exhibit altered monocyte function in the periphery and an ongoing inflammatory reaction where monocytes could be potentially recruited from the bone marrow in a CCL2-dependent manner, CCL2 measurements were performed from monocytes that were stimulated with LPS. Notably, LPS stimulation of monocytes also led to an abnormal CCL2 release by PD monocytes compared to monocytes isolated from healthy donors (Fig. 4a). Most importantly, elevated levels of CCL2 were also detected in the serum of PD patients compared to age-matched healthy controls (Fig. 4b), thereby additionally supporting the relevance of our in vitro human primary monocyte culture system. Interestingly, the gene set enrichment analysis identified CCR chemokine receptor binding as one of the top affected molecular functions as well as CCL2 as top affected signal transduction pathway (Supplementary Table 7). Matrix metalloproteinase activity, which is important for the release, movement and migration of monocytes, was also amongst the top affected pathways.

To investigate whether the observed inflammatory dysregulation is specific for PD we also included patients with other parkinsonian symptoms like patients with multiple system atrophy (MSA) and progressive supranuclear palsy (PSP) in our study. FACS-based analysis of monocytic subpopulations revealed an enrichment of classical monocytes also in PSP and MSA patients (Supplementary Fig. 5b), whereas the number of total monocytes was not significantly altered between the PD, PSP, MSA and control group (Supplementary Fig. 5a). LPS stimulation of cultured monocytes from PD patients and controls leads to an excess release of IL-6 from PD monocytes compared to controls as observed in our initial experiments. However, we also observed increased IL-6 release from PSP monocytes compared to controls. In contrast, MSA monocytes did not release excess IL-6 upon LPS stimulation (Supplementary Fig. 5c). Regarding phagocytic activity of cultured monocytes, we found only reduced phagocytic activity in PD monocytes. PSP and MSA monocytes had similar phagocytic activity than control monocytes (Supplementary Fig. 5d). We also measured CCL2 levels in the serum of PSP and MSA patients with no significant differences compared to control serum, whereas PD serum showed increased CCL2 levels (Supplementary Fig. 5e).

### FAS/FASLG system is involved in the dysregulation of monocyte subpopulations in PD patients

**Fig. 5:**
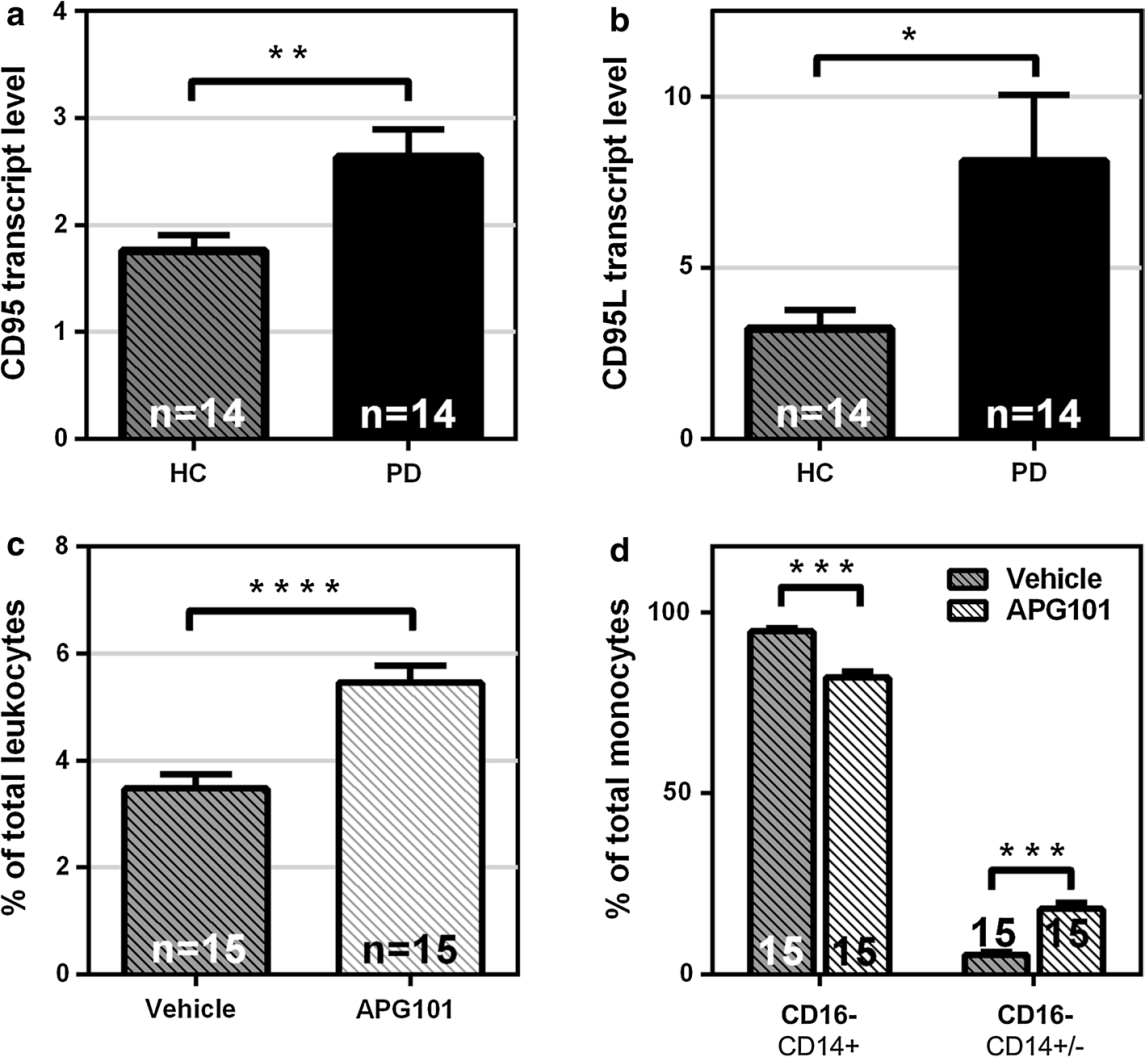
FAS/FASLG upregulation in monocytes from PD patients. The expression of both FAS (**a**) and FASLG (**b**) is upregulated in unstimulated PD monocytes (Healthy controls *n* = 14, PD patients *n* = 14). **c** Monocytes are enriched in PD leukocyte cultures after 48 h treatment with FASLG-inhibitor APG101 (*n* = 15, APG 0.25 mg/ml). **d** Classical (CD14+CD16−) monocytes are decreased and non-classical (CD14−CD16+) monocytes are increased by treatment of PD leukocytes with APG101 in culture (*n* = 15). *Bars* represent mean ± SEM. *p < 0.05, **p < 0.01, ***p < 0.001, ****p < 0.0001

Another top upstream regulator identified by the gene set enrichment analysis is FAS, which has been previously linked to inflammatory processes in mouse models of inflammation [5]. In humans and in a mouse model for spinal cord lesion, upregulation of FAS ligand (FASLG) is required for the recruitment of peripheral myeloid cells to the injured tissue [31]. To determine whether the FAS/FASLG system is involved in the peripheral dysregulation of monocytes in PD, FAS and FASLG expression levels in monocytes from PD patients were measured. Strikingly, a strong upregulation of both FAS and FASLG in monocytes from PD patients was observed (Fig. 5a, b). Importantly, deletion of myeloid FASLG or neutralization of FASLG on myeloid cells was reported to improve functional recovery after CNS trauma [31]. We, therefore, asked whether neutralization of FASLG would also have therapeutic effects on the regulation of monocyte subpopulations in PD patients. To this end, leukocytes derived from erythrocyte-depleted whole blood of PD patients was treated with the FASLG antagonist APG101 [41] for 48 h in culture. Using an FACS-based approach, we found a therapeutic effect of APG101 by a 2.5-fold reduction of the ratio of classical to non-classical monocytes derived from PD patients compared to vehicle-treated cells (Fig. 5d). The same reduction of the ratio of classical to non-classical monocytes was observed for healthy controls (Supplementary Fig. 6).

## Discussion

Using a multimodal approach, we identified a dysregulation of peripheral human monocytes in PD. We have shown that PD monocytes have a pro-inflammatory predisposition based on their transcriptome profile and disbalance of monocytic subpopulations. The increased ratio in pro-inflammatory classical monocytes in PD patients was accompanied by an activation of the CCR2–CCL2 axis in PD. Moreover, using a human primary monocyte culture system we were able to demonstrate that PD monocytes respond to a “second hit” with hyperactivation, which correlated with clinical disease stages. Finally, we demonstrate that the FAS/FASLG system is essentially involved in deregulation of monocyte subpopulations, which can be pharmacologically reverted by neutralization of FASLG.

In the transcriptome analysis, we found that the response to inflammatory triggers is dysregulated on a transcriptional level in PD. This dysregulation could also be confirmed in an independent cohort on the basis of the single genes JUN, FOSB, EGR1, DUSP1, RHOB, FOS and NFKBIZ. Thus, PD monocytes were preconditioned to behave abnormally upon inflammatory stimulation. However, our work was not designed as a classical biomarker study. We did not investigate the correlation of mRNA levels of specific genes in monocytes and disease severity. This would possibly require larger and or more homogenous PD patient cohorts including, for example, the exclusion of specific mutations in PARK genes. Nevertheless, the findings of this study could be the basis for the development of easily accessible PD biomarkers in the blood of PD patients and might turn out to be valuable readouts to judge future clinical studies on disease-modifying therapies. In a complementary approach using a FACS-based technology, we were able to confirm a pro-inflammatory dysregulation of monocytic subpopulations.

These primary abnormalities of PD monocytes suggest that monocytes from PD patients also behave functionally different, supported by an impaired phagocytic function of PD monocytes (this study and [23]). Since monocyte-derived CNS macrophages can contribute to the resolution of neuroinflammation by clearing of cell debris and extracellular material [19], a decrease of phagocytic activity could be detrimental. Additional proof for functional abnormalities of monocytes in PD patients comes from our primary human monocyte culture system. Unlike the study of Hasegawa et al. [27], we found functional overactivity upon LPS stimulation of cultured monocytes from PD patients compared to healthy controls. The discrepancy to the study of Hasegawa et al. who found lower levels of certain cytokines (IL-1β, TNFα, IL-6 and IFNγ) released by PBMCs might be explained by the monocyte isolation method. While Hasegawa et al. used gradient density sedimentation leading ultimately to the isolation of monocytes and lymphocytes (contained in the PBMC fraction), we used positive magnetic selection based on CD14 expression leading to the isolation of solely monocytes. Additionally, when Hasegawa et al. enriched monocytes in culture by changing the media they could not confirm decreased cytokine release in their cultures suggesting that the lymphocytes contained in the culture of Hasegawa et al. and the isolation method account for the discrepancy of the two studies. Strikingly, increased cytokine secretion from PD monocytes upon LPS stimulation correlated in our study with PD disease state, thereby suggesting that monocyte hyperactivation is linked to disease severity. Collectively, our findings indicate that myeloid cells of the innate immune system are functionally altered and point to a pro-inflammatory predisposition of the innate immune system of PD patients.

Most importantly, PD monocytes were not functionally overactive a priori but required a “second hit” to exert their abnormal activity. Such “second hits” could be environmental cues or CNS factors triggering the peripheral immune system. Strong support for this theory comes from the study of Gao et al. [22], who showed that LPS-induced inflammation and alpha-synuclein pathology act synergistically and potentiate neurodegeneration in a genetic mouse model of PD. Similarly, recent studies demonstrated that repeated LPS injections exacerbated the pathogenesis of ALS in a mouse model system [33] and impaired the behavioral phenotype in prion-diseased animals [14]. Our results suggest that the progression or development of neurological diseases might be affected by the number and type of environmental second hits in conjunction with an existing predisposition. Our data from PD monocytes and previous experimental evidence are complemented by the association of influenza infections, asthma and allergic rhinitis to the development of PD [7, 26]. Our data might also provide an explanation for the clinical observation that PD patients often do not fully recover from worsened PD symptoms after severe systemic infections. However, further studies are needed to translate our findings to clinical therapies.

The increase of classical CD14+CD16− monocytes and decrease in the non-classical CD14−CD16+ monocytes in the blood of PD patients observed in this study could result from both differentiation of monocytes and monocyte precursors to the classical pro-inflammatory phenotype or from increased migration from the bone marrow. Support for the latter comes from our observation of elevated levels of CCL2 in the serum of PD patients, a classical signal for recruitment of monocytes from the bone marrow [37]. In agreement with this idea, Funk et al. demonstrated an upregulation of surface CCL2 receptor (CCR2) on classical monocytes of PD patients [21]. The elevated serum levels of CCL2 in PD could possibly be attributed to the increased production of the chemokine by monocytes themselves. In line with this, we found that upon LPS stimulation, PD monocytes secrete significantly higher levels of CCL2 compared to control monocytes. Together, these results suggest that the CCR2–CCL2 axis ranging from CCL2 release to altered composition of monocytes is activated in PD. These results are also in agreement with previous studies [6, 35] demonstrating elevated LPS-induced CCL2 production from PD monocytes. CCL2 release by monocytes could be a part of a feedback loop serving to enhance the further recruitment of monocytes to the inflammatory site. Interestingly, activated microglia can also secrete CCL2 [16], which can cross the blood–brain barrier and recruit monocytes into the inflamed CNS. Reactive microgliosis has been observed in the affected PD brain regions and is hypothesized to be triggered by extracellular alpha-synuclein and apoptotic neurons [25]. Thus, the increased CCL2 serum levels and increase of classical monocytes in our PD cohort could possibly also be a result of the secretion of inflammatory mediators by microglia.

To gain more insight in the specificity of the observed dysregulation of peripheral human monocytes in PD, we also included PSP and MSA patients as another disease cohort with parkinsonian symptoms. Overall, we found evidence for inflammatory dysregulation also in PSP and MSA with regard to some aspects (enrichment of classical monocytes, increased IL-6 release upon LPS stimulation for the PSP cohort), while other changes are clearly specific for PD (decreased phagocytic activity, increased CCL2 serum levels for MSA). However, it has to be taken into account that due to the limited number of MSA and PSP patients a definitive statement regarding inflammatory dysregulation in PSP and MSA remains difficult. Further studies including larger patient numbers for MSA and PSP are needed to fully understand the biology of peripheral monocytes in MSA and PSP.

Three pieces of evidence in our study suggest an essential involvement of the FAS/FASLG system in the dysregulation of PD monocytes. First, we found a strong upregulation of both FAS and FASLG in monocytes from PD patients. Second, the transcriptome gene set enrichment analysis identified FAS as a top upstream regulator of dysregulated genes in PD monocytes. Finally, pharmacological neutralization of FASLG could reverse the increased ratio of classical to non-classical monocytes in PD patients and controls in vitro suggesting that the FAS/FASLG system plays a crucial role in the regulation of human monocyte subpopulations. Our results are also in line with the findings of Brochard et al. [8] describing an FASLG-dependent dopaminergic toxicity in MPTP-treated mice. Neutralization of FASLG could hold a therapeutic promise, and clinical studies based on the FASLG neutralizing fusion protein used in our study (APG101) are clearly warranted. Importantly, APG101 has already been successfully applied to patients and turned out to be safe in a neuro-oncological context [41]. However, further studies are needed to fully translate our findings into clinical therapies.

In summary, our findings provide the first direct evidence that circulating myeloid cells are altered in terms of their transcriptome profile, subset composition, and function in PD patients. Furthermore, we present the FAS/FASLG system as a regulator of monocyte subpopulations which could be targeted in pharmacological approaches to treat PD. A better understanding of monocyte function and the role of the FAS/FASLG system as well as first safety and proof-of-principle studies in PD patients using APG101 may pave the way to novel therapies aimed at halting disease progression in PD patients.

## Electronic supplementary material

Below is the link to the electronic supplementary material.

Supplementary material 1 (DOCX 114 kb)

Supplementary material 2 (DOCX 426 kb)

Supplementary material 3 (DOCX 246 kb)

Supplementary material 4 (DOCX 520 kb)

Supplementary material 5 (DOCX 208 kb)

Supplementary material 6 (DOCX 100 kb)

Supplementary material 7 (DOCX 19 kb)

Supplementary material 8 (DOCX 21 kb)

Supplementary material 9 (DOCX 25 kb)

Supplementary material 10 (DOCX 25 kb)

Supplementary material 11 (DOCX 18 kb)

Supplementary material 12 (DOCX 12 kb)

Supplementary material 13 (DOCX 15 kb)

Supplementary material 14 (DOCX 12 kb)
