# Supplementary material for: Inflammatory dysregulation of blood monocytes in Parkinson’s disease patients
Source: Acta Neuropathol. 2014 Oct 5;128(5):651–63. doi: 10.1007/s00401-014-1345-4 (PMC4201759; doi:10.1007/s00401-014-1345-4)
Supplement: Supplementary file 1 — Supplementary material 1 (DOCX 114 kb) [file 401_2014_1345_MOESM1_ESM.docx]

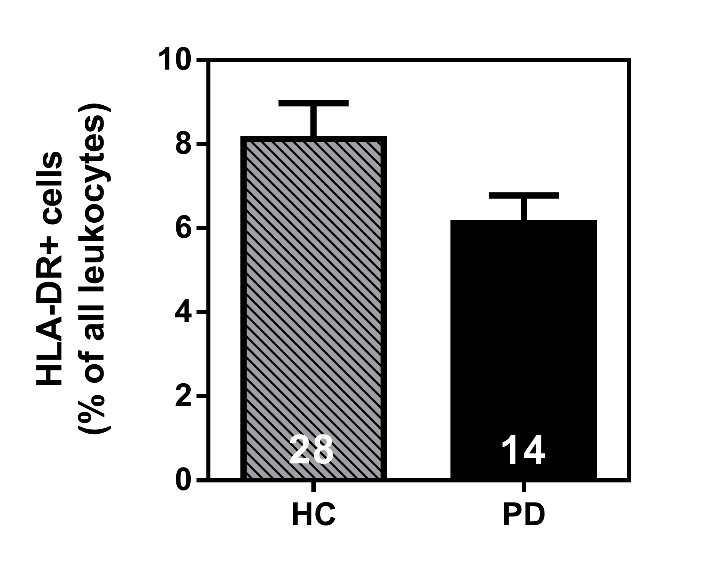


**Supplementary Figure 1.** Quantification of HLA-DR+ cells as percent of all leukocytes in the blood of healthy controls (n=28) and PD patients (n=14)
