# Supplementary material for: Inflammatory dysregulation of blood monocytes in Parkinson’s disease patients
Source: Acta Neuropathol. 2014 Oct 5;128(5):651–63. doi: 10.1007/s00401-014-1345-4 (PMC4201759; doi:10.1007/s00401-014-1345-4)
Supplement: Supplementary file 2 — Supplementary material 2 (DOCX 426 kb) [file 401_2014_1345_MOESM2_ESM.docx]

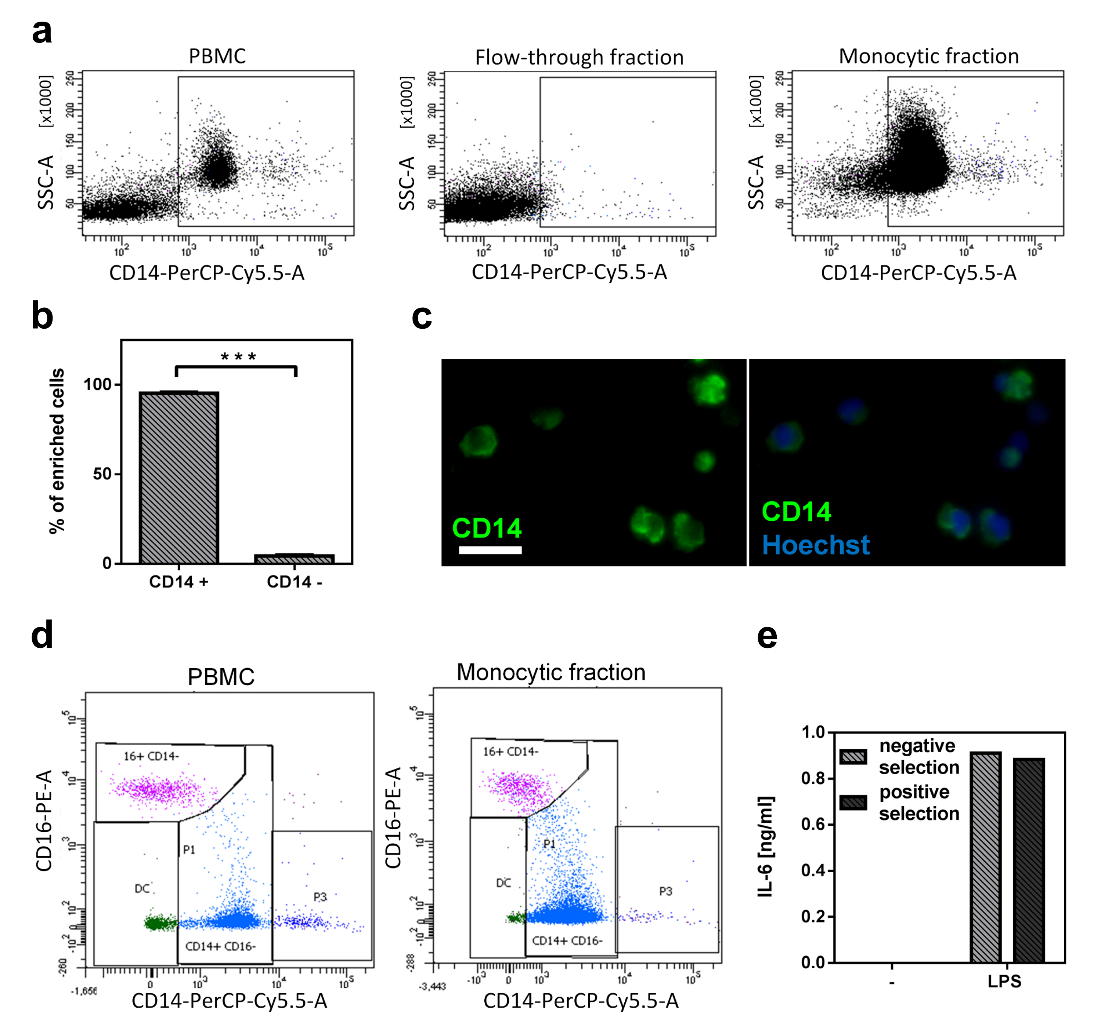


**Supplementary Figure 2**

**Characterization of monocytes after magnetic bead separation.** **(a)** Representative FACS analysis of peripheral blood mononuclear cells (PBMC) before magnetic separation (left panel) and after separation (non-monocytes:- middle panel; monocytic fraction: right panel). **(b)** Quantification of CD14-postive cells after magnetic separation. The monocyte-enriched fraction contains 95% CD14+ cells (n = 3, ***p<0.001) **(c)** Cells in the monocytic fraction stain positive for anti-CD14 (green). Scale bar: 50 µm **(d)** Before (PBMC) and after magnetic bead separation both classical and non-classical monocytes are contained in the monocytic fraction (monocytes). **(e)** Monocytes isolated by both positive (aCD14) and negative (untouched) selection are not activated by the separation. Positive selection with anti-CD14 magnetic beads does not interfere with LPS (1 ng/ml) stimulation.
