# Supplementary material for: Inflammatory dysregulation of blood monocytes in Parkinson’s disease patients
Source: Acta Neuropathol. 2014 Oct 5;128(5):651–63. doi: 10.1007/s00401-014-1345-4 (PMC4201759; doi:10.1007/s00401-014-1345-4)
Supplement: Supplementary file 3 — Supplementary material 3 (DOCX 246 kb) [file 401_2014_1345_MOESM3_ESM.docx]

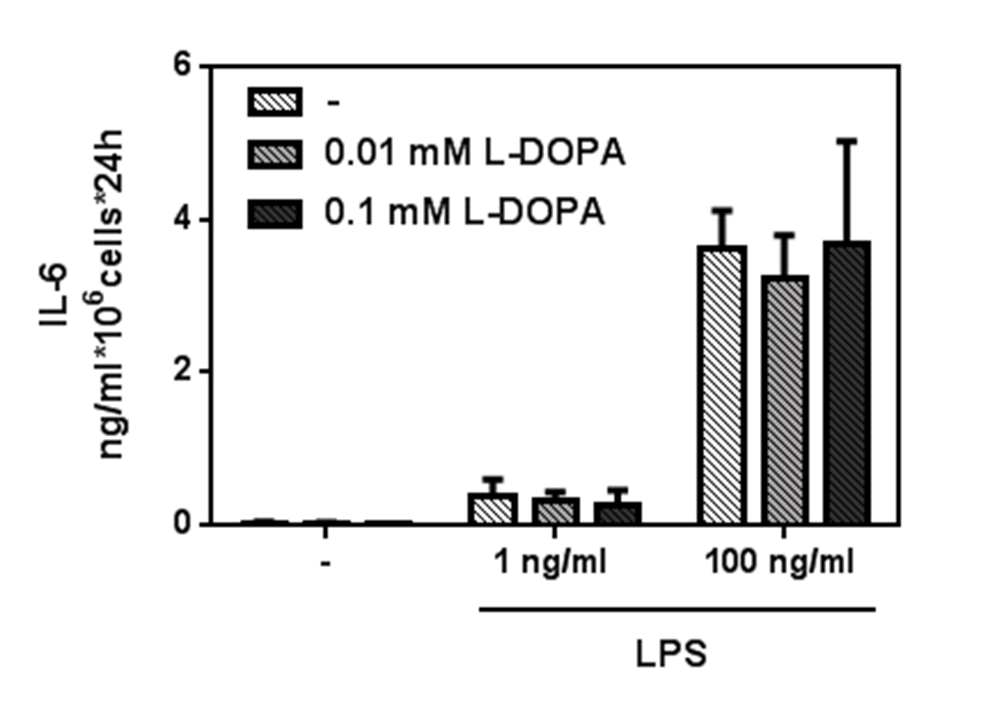


**Supplementary Figure 3**

**L-Dopa supplementation does not increase IL-6 release.** ELISA quantification of IL-6 levels in the conditioned media from monocytes of healthy controls (n = 3) does not show significant differences upon treatment with physiological L-Dopa levels. Bars represent mean ± SEM.
