# Supplementary material for: Inflammatory dysregulation of blood monocytes in Parkinson’s disease patients
Source: Acta Neuropathol. 2014 Oct 5;128(5):651–63. doi: 10.1007/s00401-014-1345-4 (PMC4201759; doi:10.1007/s00401-014-1345-4)
Supplement: Supplementary file 4 — Supplementary material 4 (DOCX 520 kb) [file 401_2014_1345_MOESM4_ESM.docx]

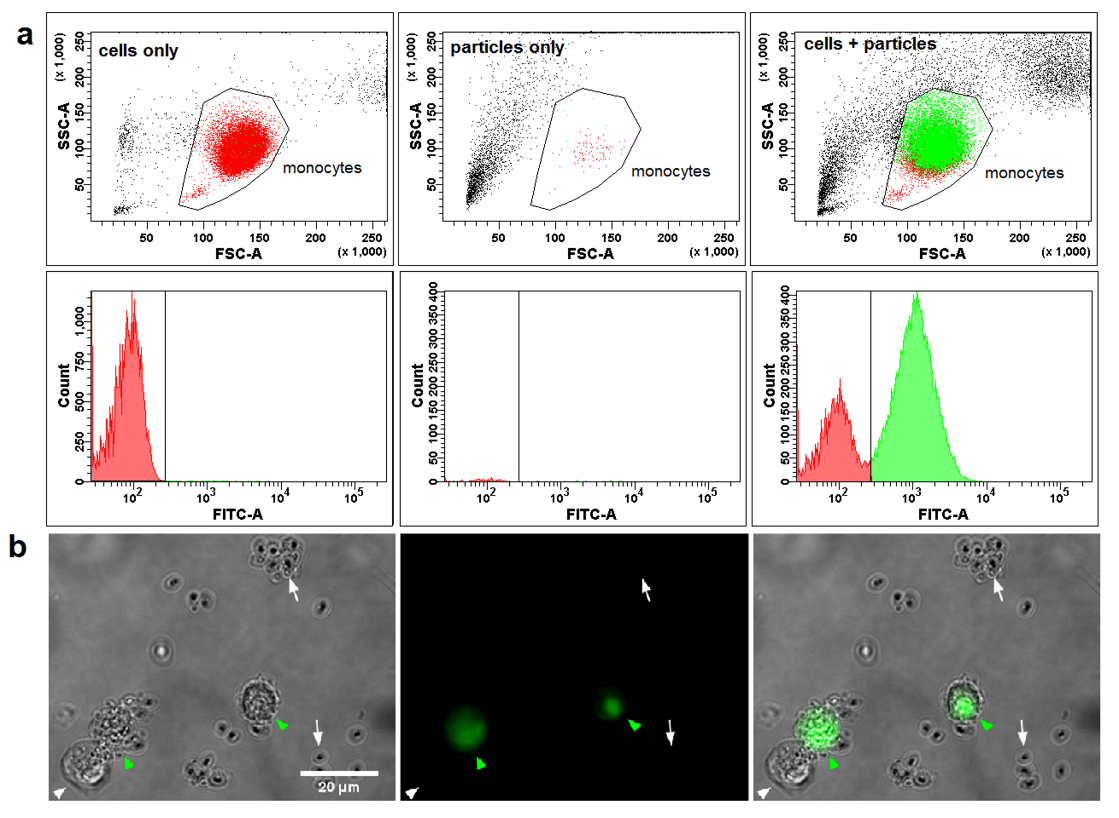


**Supplementary Figure 4. FACS-based (a) and microscope-based (b) analysis of uptake of PhRodo™- labelled yeast wall particles.** Phagocytosis by monocytes can be detected by the increase of particle fluorescence in acidic compartments. White arrowheads indicate non-phagocytizing cells, green arrowheads indicate phagocytizing cells and white arrows indicate non-engulfed particles. Scale bar: 20 µm.
