# Supplementary material for: Inflammatory dysregulation of blood monocytes in Parkinson’s disease patients
Source: Acta Neuropathol. 2014 Oct 5;128(5):651–63. doi: 10.1007/s00401-014-1345-4 (PMC4201759; doi:10.1007/s00401-014-1345-4)
Supplement: Supplementary file 5 — Supplementary material 5 (DOCX 208 kb) [file 401_2014_1345_MOESM5_ESM.docx]

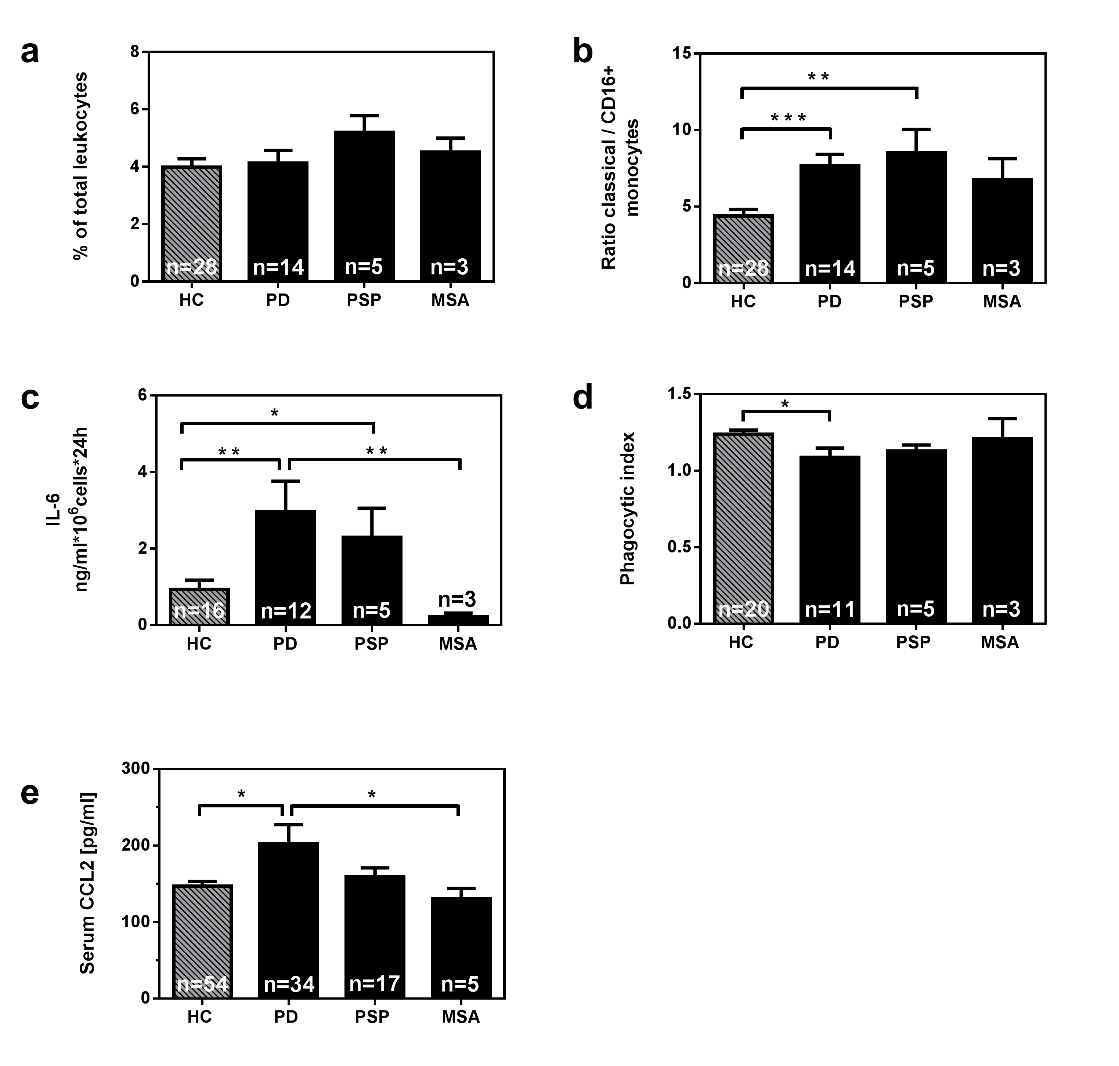


**Supplementary Figure 5. Functional characterization of monocytes from patients with PSP and MSA.** **a,** total monocyte numbers and **b,** ratio of classical CD14+CD16- monocytes to CD14+/-CD16+ monocytes. **c**, IL-6 release after stimulation with LPS (1ng/ml). **d,** Phagocytic activity of monocytes from healthy controls and PD/PSP/MSA patients. **e,** serum levels of CCL2 are similar in healthy controls, PSP and MSA patients and elevated in PD patients. Bars = mean±SEM, *p<0.05, **p<0.01, ***p<0.001.
