# Supplementary material for: Inflammatory dysregulation of blood monocytes in Parkinson’s disease patients
Source: Acta Neuropathol. 2014 Oct 5;128(5):651–63. doi: 10.1007/s00401-014-1345-4 (PMC4201759; doi:10.1007/s00401-014-1345-4)
Supplement: Supplementary file 6 — Supplementary material 6 (DOCX 100 kb) [file 401_2014_1345_MOESM6_ESM.docx]

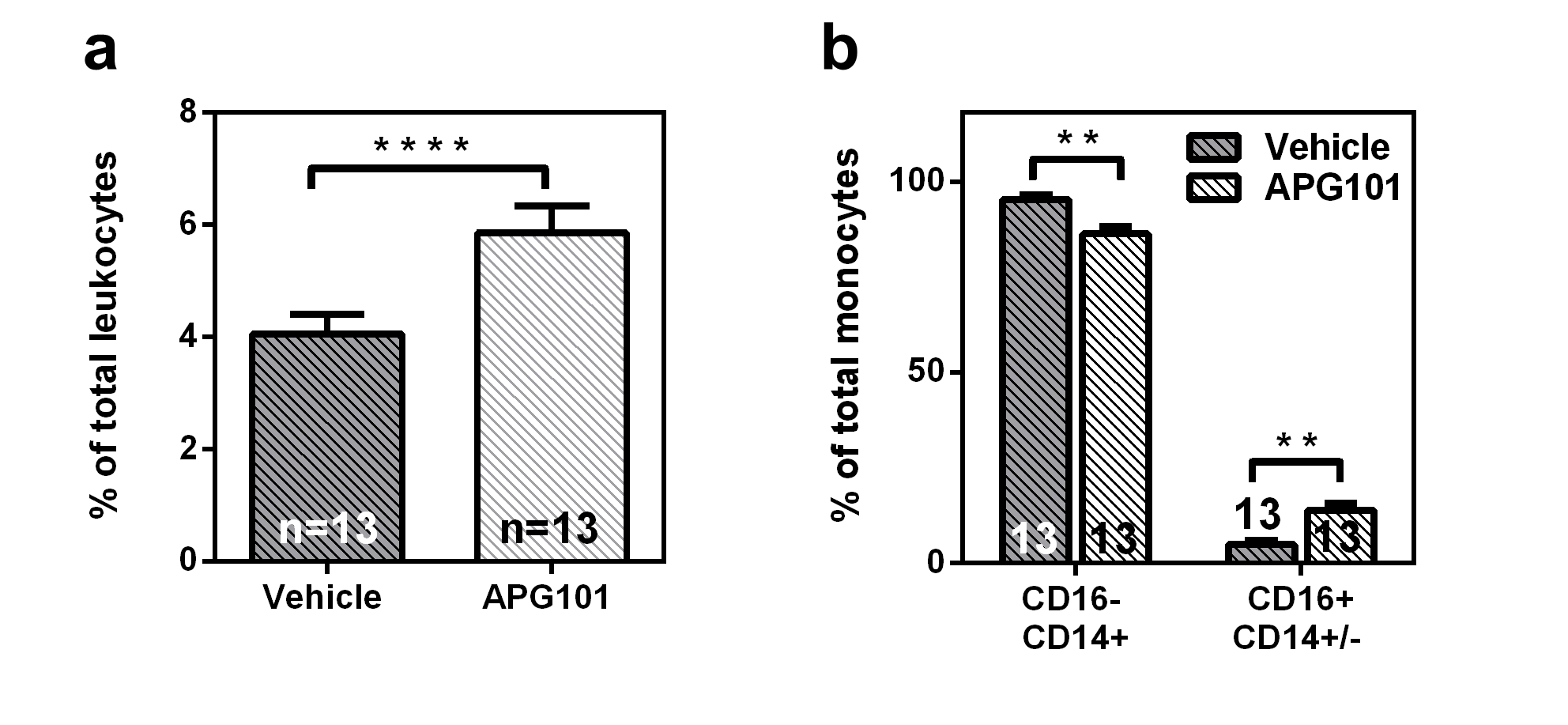


**Supplementary Figure 6**

**FASLG neutralization in monocyte culture from healthy controls.** **(a)** Monocytes are enriched in healthy control leukocyte culture after 48 h treatment with FASLG inhibitor APG101 (n = 12, APG 0.25 mg/ml). **(b)** Classical (CD14+CD16-) monocytes are decreased and non-classical monocytes are increased by treatment of healthy controls’ leukocytes with APG101 in culture (n=13). Bars represent mean ± SEM, **p<0.01, ****p<0.0001.
