# Supplementary material for: Inflammatory dysregulation of blood monocytes in Parkinson’s disease patients
Source: Acta Neuropathol. 2014 Oct 5;128(5):651–63. doi: 10.1007/s00401-014-1345-4 (PMC4201759; doi:10.1007/s00401-014-1345-4)
Supplement: Supplementary file 7 — Supplementary material 7 (DOCX 19 kb) [file 401_2014_1345_MOESM7_ESM.docx]

| **SUPPLEMENTARY TABLE 1. Characteristics of human cases for analysis of monocyte subpolulations (cohort Fig. 1)** | | | | | | |
| --- | --- | --- | --- | --- | --- | --- |
| **ID** | **gender** | **age** | **age of onset** | **disease duration [y]** | **medication** | **co-morbidities** |
| **PD #1** | m | 62 | N/K | N/K | DA agonist, MAO inhibitor | N/K |
| **PD #2** | m | 75 | 68 | 7 | L-dopa, DA agonist | bladder emptying problems, arterial hypertension, diabetes mellitus type II |
| **PD #3** | f | 78 | 63 | 15 | L-dopa, DA agonist | dementia |
| **PD #4** | m | 69 | N/K | N/K | no | N/K |
| **PD #5** | m | 49 | 44 | 5 | DA agonist, MAO inhibitor | depression |
| **PD #6** | f | 60 | 58 | 2 | L-dopa, DA agonist | swell of spinal disk, adiposity |
| **PD #7** | m | 80 | 66 | 14 | L-dopa, DA agonist, MAO inhibitor, AChE inhibitor | osteoporosis, anemia |
| **PD #8** | m | 66 | 49 | 17 | L-dopa | hyponatremia |
| **PD #9** | m | 65 | 59 | 6 | L-dopa, DA agonist, MAO inhibitor | dementia, diabetes mellitus type II |
| **PD #10** | m | 68 | 64 | 4 | DA agonist, MAO inhibitor | no |
| **PD #11** | f | 76 | 66 | 10 | L-dopa | arterial hypertension |
| **PD #12** | m | 68 | 58 | 10 | L-dopa, DA agonist | coronary heart disease, arterial hypertension, lactose intolerance |
| **PD #13** | m | 73 | 61 | 12 | L-dopa, DA agonist | arterial hypertension |
| **PD #14** | m | 68 | 58 | 10 | L-dopa | esophagitis, degenerative lumbar spine changes |
| **ID** | **gender** | **age** | **age of onset** | **disease duration [y]** | **medication** | **co-morbidities** |
| **Ctrl #1** | m | 58 | N/A | N/A | N/A | N/A |
| **Ctrl #2** | m | 71 | N/A | N/A | N/A | N/A |
| **Ctrl #3** | m | 70 | N/A | N/A | N/A | N/A |
| **Ctrl #4** | f | 76 | N/A | N/A | N/A | N/A |
| **Ctrl #5** | m | 71 | N/A | N/A | N/A | N/A |
| **Ctrl #6** | m | 71 | N/A | N/A | N/A | N/A |
| **Ctrl #7** | f | 74 | N/A | N/A | N/A | N/A |
| **Ctrl #8** | m | 78 | N/A | N/A | N/A | N/A |
| **Ctrl #9** | f | 87 | N/A | N/A | N/A | N/A |
| **Ctrl #10** | f | 79 | N/A | N/A | N/A | N/A |
| **Ctrl #11** | m | 81 | N/A | N/A | N/A | N/A |
| **Ctrl #12** | f | 61 | N/A | N/A | N/A | N/A |
| **Ctrl #13** | m | 66 | N/A | N/A | N/A | N/A |
| **Ctrl #14** | m | 78 | N/A | N/A | N/A | N/A |
| **Ctrl #15** | m | 70 | N/A | N/A | N/A | N/A |
| **Ctrl #16** | m | 73 | N/A | N/A | N/A | N/A |
| **Ctrl #17** | f | 65 | N/A | N/A | N/A | N/A |
| **Ctrl #18** | f | 72 | N/A | N/A | N/A | N/A |
| **Ctrl #19** | m | 61 | N/A | N/A | N/A | N/A |
| **Ctrl #20** | f | 69 | N/A | N/A | N/A | N/A |
| **Ctrl #21** | f | 72 | N/A | N/A | N/A | N/A |

The table summarizes the characteristics of PD patients and controls (Ctrl) from Ulm University. N/K= not known; N/A=not applicable, DA=dopamin, MAO=monoaminooxidase, AChE= acetylcholinesterase
