# Supplementary material for: Inflammatory dysregulation of blood monocytes in Parkinson’s disease patients
Source: Acta Neuropathol. 2014 Oct 5;128(5):651–63. doi: 10.1007/s00401-014-1345-4 (PMC4201759; doi:10.1007/s00401-014-1345-4)
Supplement: Supplementary file 8 — Supplementary material 8 (DOCX 21 kb) [file 401_2014_1345_MOESM8_ESM.docx]

| **SUPPLEMENTARY TABLE 2a. Characteristics of human discovery cohort next generation sequencing study(cohort Fig. 2a-d)** | | | | | | |
| --- | --- | --- | --- | --- | --- | --- |
| **ID** | **gender** | **age** | **age of onset** | **disease duration [y]** | **medication** | **co-morbidities** |
| **PD #15** | m | 75 | 66 | 9 | L-dopa | prostate carcinoma (> 5 years ago) |
| **PD #16** | f | 73 | 45 | 28 | L-dopa | vitamin B12 deficiency |
| **PD #17** | m | 77 | 67 | 10 | L-dopa | prostate carcinoma (> 5 years ago) |
| **PD #18** | m | 72 | 68 | 4 | L-dopa, DA agonist | hyperthyreoses, glaucoma |
| **PD #19** | m | 74 | 60 | 14 | L-dopa | depression, prostate hyperplasia |
| **PD #20** | m | 79 | 67 | 12 | L-dopa | Meniere's disease |
| **PD #21** | f | 59 | 51 | 8 | L-dopa | adiposity |
| **PD #22** | m | 72 | 64 | 8 | L-dopa, DA agonist | arterial hypertension |
| **ID** | **gender** | **age** | **age of onset** | **disease duration [y]** | **medication** | **co-morbidities** |
| **Ctrl #22** | m | 82 | N/A | N/A | N/A | N/A |
| **Ctrl #23** | m | 70 | N/A | N/A | N/A | N/A |
| **Ctrl #24** | m | 69 | N/A | N/A | N/A | N/A |
| **Ctrl #25** | f | 68 | N/A | N/A | N/A | N/A |
| **Ctrl #26** | f | 87 | N/A | N/A | N/A | N/A |
| **Ctrl #27** | f | 71 | N/A | N/A | N/A | N/A |
| **Ctrl #28** | f | 70 | N/A | N/A | N/A | N/A |
| **Ctrl #29** | f | 62 | N/A | N/A | N/A | N/A |
| **Ctrl #30** | f | 60 | N/A | N/A | N/A | N/A |

| **TABLE 2b. Characteristics of human validation cohort (cohort Fig. 2e and Figure 5 a, b)** | | | | | | |
| --- | --- | --- | --- | --- | --- | --- |
| **ID** | **gender** | **age** | **age of onset** | **disease duration [y]** | **medication** | **co-morbidities** |
| **PD#41** | m | 71 | 57 | 14 | L-dopa | depression, sleep apnea |
| **PD#42** | f | 81 | 77 | 4 | L-dopa | dementia, adiposity, diabetes mellitus type II |
| **PD#39** | f | 61 | 51 | 10 | L-dopa, DA agonist | adiposity, arterial hypertension, aortic insufficiency |
| **PD#40** | f | 61 | 51 | 10 | L-dopa, DA agonist | arterial hypertension, disc prolapse |
| **PD#43** | f | 78 | 64 | 14 | L-dopa, DA agonist, MAO inhibitor | restless legs syndrome, degeneration of macula |
| **PD#44** | m | 63 | 52 | 11 | L-dopa | arterial hypertension |
| **PD#11** | f | 76 | 66 | 10 | L-dopa | arterial hypertension |
| **PD#1** | m | 62 | N/K | N/K | DA agonist, MAO inhibitor | N/K |
| **PD#2** | m | 75 | 68 | N/K | L-dopa, DA agonist | bladder emptying problems, arterial hypertension, diabetes mellitus type II |
| **PD#4** | m | 69 | N/K | N/K | no | N/K |
| **PD#38** | f | 67 | 52 | 15 | L-dopa, DA agonist | plexus lesion, arterial hypertension |
| **PD#3** | f | 78 | 63 | 15 | L-dopa, DA agonist | dementia |
| **PD#5** | m | 49 | 44 | 5 | DA agonist, MAO inhibitor | depression |
| **PD#6** | f | 60 | 58 | 2 | L-dopa, DA agonist | swell of spinal disk, adiposity |
| **ID** | **gender** | **age** | **age of onset** | **disease duration [y]**6 | **medication** | **co-morbidities** |
| **Ctrl#1** | m | 58 | N/A | N/A | N/A | N/A |
| **Ctrl#2** | m | 71 | N/A | N/A | N/A | N/A |
| **Ctrl#3** | m | 70 | N/A | N/A | N/A | N/A |
| **Ctrl#4** | f | 76 | N/A | N/A | N/A | N/A |
| **Ctrl#6** | m | 71 | N/A | N/A | N/A | N/A |
| **Ctrl#7** | f | 74 | N/A | N/A | N/A | N/A |
| **Ctrl#8** | m | 78 | N/A | N/A | N/A | N/A |
| **Ctrl#9** | f | 87 | N/A | N/A | N/A | N/A |
| **Ctrl#10** | f | 79 | N/A | N/A | N/A | N/A |
| **Ctrl#11** | m | 81 | N/A | N/A | N/A | N/A |
| **Ctrl#13** | m | 66 | N/A | N/A | N/A | N/A |
| **Ctrl#15** | m | 70 | N/A | N/A | N/A | N/A |
| **Ctrl#17** | f | 65 | N/A | N/A | N/A | N/A |
| **Ctrl#43** | m | 83 | N/A | N/A | N/A | N/A |

The table summarizes the characteristics of PD patients and controls (Ctrl) from Ulm University. N/K= not known; N/A=not applicable, DA=dopamin, MAO=monoaminooxidase
