# Supplementary material for: Inflammatory dysregulation of blood monocytes in Parkinson’s disease patients
Source: Acta Neuropathol. 2014 Oct 5;128(5):651–63. doi: 10.1007/s00401-014-1345-4 (PMC4201759; doi:10.1007/s00401-014-1345-4)
Supplement: Supplementary file 9 — Supplementary material 9 (DOCX 25 kb) [file 401_2014_1345_MOESM9_ESM.docx]

| **SUPPLEMENTARY TABLE 3a. Characteristics of PD patients (cohort Fig. 3a, c-f and Figure 4a)** | | | | | | |
| --- | --- | --- | --- | --- | --- | --- |
| **ID** | **gender** | **age** | **age of onset** | **disease duration [y]** | **medication** | **co-morbidities** |
| **PD#23** | m | 72 | 62 | 10 | L-dopa, DA agonist | no |
| **PD#24** | f | 72 | 60 | 12 | L-dopa, DA agonist, AChE inhibitor, SSRI | mamma carcinoma (>5 years ago) |
| **PD#25** | m | 75 | 59 | 16 | L-dopa | phimosis, kyphosis |
| **PD#26** | f | 78 | 65 | 13 | L-dopa | depression, dementia |
| **PD#27** | f | 66 | 45 | 21 | L-dopa | depression |
| **PD#28** | f | 78 | 68 | 10 | L-dopa | no |
| **PD#29** | m | 74 | 62 | 12 | L-dopa | no |
| **PD#30** | m | 77 | 64 | 13 | L-dopa | depression, sleep apnea |
| **PD#31** | m | 76 | 73 | 3 | no | depression |
| **PD#32** | f | 79 | 68 | 11 | L-dopa | dementia, depression |
| **ID** | **gender** | **age** | **age of onset** | **disease duration [y]** | **medication** | **co-morbidities** |
| **Ctrl#31** | f | 75 | N/A | N/A | N/A | N/A |
| **Ctrl#32** | f | 62 | N/A | N/A | N/A | N/A |
| **Ctrl#33** | m | 80 | N/A | N/A | N/A | N/A |
| **Ctrl#34** | m | 70 | N/A | N/A | N/A | N/A |
| **Ctrl#35** | m | 71 | N/A | N/A | N/A | N/A |
| **Ctrl#36** | f | 74 | N/A | N/A | N/A | N/A |
| **Ctrl#37** | m | 62 | N/A | N/A | N/A | N/A |
| **Ctrl#17** | f | 65 | N/A | N/A | N/A | N/A |
| **Ctrl#38** | f | 51 | N/A | N/A | N/A | N/A |
| **Ctrl#39** | m | 59 | N/A | N/A | N/A | N/A |

| **SUPPLEMENTARY TABLE 3b. Characteristics of PD patients (cohort Fig. 3b, h)** | | | | | | |
| --- | --- | --- | --- | --- | --- | --- |
| **ID** | **gender** | **age** | **age of onset** | **disease duration [y]** | **medication** | **co-morbidities** |
| **PD#24** | f | 72 | 60 | 12 | L-dopa, DA agonist, AChE inhibitor, SSRI | mamma carcinoma (>5 years ago) |
| **PD#25** | m | 75 | 59 | 16 | L-dopa | phimosis, kyphosis |
| **PD#26** | f | 78 | 65 | 13 | L-dopa | depression, dementia |
| **PD#27** | f | 66 | 45 | 21 | L-dopa | depression |
| **PD#28** | f | 78 | 68 | 10 | L-dopa | no |
| **PD#30** | m | 77 | 64 | 13 | L-dopa | depression, sleep apnea |
| **PD#31** | m | 76 | 73 | 3 | no | depression |
| **PD#33** | f | 66 | 53 | 13 | L-dopa, DA agonist, NMDA agonist, MAO inhibitor, SSRI | coronary heart disease |
| **PD#34** | m | 74 | 69 | 5 | L-dopa | no |
| **PD#35** | f | 73 | 67 | 6 | L-dopa, DA agonist, MAO inhibitor | chronic kidney insufficiency, arterial hypertension, diabetes mellitus type II |
| **PD#36** | m | 74 | 68 | 6 | L-dopa, DA agonist, AChE inhibitor | depression, arterial hypertension |
| **PD#37** | m | 69 | 59 | 10 | L-dopa, DA agonist, MAO inhibitor | no |
| **PD#32** | f | 79 | 68 | 11 | L-dopa | dementia, depression |
| **PD#4** | m | 69 | N/K | N/K | N/K | N/K |
| **PD#38** | f | 67 | 52 | 15 | L-dopa, DA agonist | plexus lesion, arterial hypertension |
| **PD #6** | f | 60 | 58 | 2 | L-dopa, DA agonist | swell of spinal disk, adiposity |
| **ID** | PD#36 | **age** | **age of onset** | **disease duration [y]** | **medication** | **co-morbidities** |
| **Ctrl#31** | f | 75 | N/A | N/A | N/A | N/A |
| **Ctrl#32** | f | 62 | N/A | N/A | N/A | N/A |
| **Ctrl#33** | m | 80 | N/A | N/A | N/A | N/A |
| **Ctrl#34** | m | 70 | N/A | N/A | N/A | N/A |
| **Ctrl#35** | m | 71 | N/A | N/A | N/A | N/A |
| **Ctrl#36** | f | 74 | N/A | N/A | N/A | N/A |
| **Ctrl#37** | m | 62 | N/A | N/A | N/A | N/A |
| **Ctrl#17** | f | 65 | N/A | N/A | N/A | N/A |
| **Ctrl#38** | f | 51 | N/A | N/A | N/A | N/A |
| **Ctrl#39** | m | 59 | N/A | N/A | N/A | N/A |

| **SUPPLEMENTARY TABLE 3c. Characteristics of PD patients (cohort Fig. 3i)** | | | | | | |
| --- | --- | --- | --- | --- | --- | --- |
| **ID** | **gender** | **age** | **age of onset** | **disease duration [y]** | **medication** | **co-morbidities** |
| **PD#15** | m | 75 | 66 | 9 | L-dopa | prostate carcinoma (> 5 years ago) |
| **PD#16** | f | 73 | 45 | 28 | L-dopa | vitamin B12 deficiency |
| **PD#17** | m | 77 | 67 | 10 | L-dopa | prostate carcinoma (> 5 years ago) |
| **PD#18** | m | 72 | 68 | 4 | L-dopa, DA agonist | hyperthyreoses, glaucoma |
| **PD#19** | m | 74 | 60 | 14 | L-dopa | depression, prostate hyperplasia |
| **PD#20** | m | 79 | 67 | 12 | L-dopa | Meniere's disease |
| **PD#21** | f | 59 | 51 | 8 | L-dopa | adiposity |
| **PD#22** | m | 72 | 64 | 8 | L-dopa, DA agonist | arterial hypertension |
| **PD#55** | m | 74 | N/K | N/K | L-dopa, DA agonist | prostate carcinoma (> 5 years ago), Vitamin B12 deficiency |
| **PD#56** | m | 46 | N/K | N/K | DA agonist | no |
| **PD#57** | m | 69 | N/K | N/K | L-dopa | N/K |
| **ID** | **gender** | **age** | **age of onset** | **disease duration [y]** | **medication** | **co-morbidities** |
| **Ctrl#22** | m | 82 | N/A | N/A | N/A | N/A |
| **Ctrl#23** | m | 70 | N/A | N/A | N/A | N/A |
| **Ctrl#24** | m | 69 | N/A | N/A | N/A | N/A |
| **Ctrl#25** | f | 68 | N/A | N/A | N/A | N/A |
| **Ctrl#26** | f | 87 | N/A | N/A | N/A | N/A |
| **Ctrl#29** | f | 62 | N/A | N/A | N/A | N/A |
| **Ctrl#30** | f | 60 | N/A | N/A | N/A | N/A |
| **Ctrl#66** | m | 82 | N/A | N/A | N/A | N/A |
| **Ctrl#67** | f | 72 | N/A | N/A | N/A | N/A |
| **Ctrl#68** | f | 56 | N/A | N/A | N/A | N/A |
| **Ctrl#69** | m | 75 | N/A | N/A | N/A | N/A |
| **Ctrl#70** | m | 61 | N/A | N/A | N/A | N/A |
| **Ctrl#71** | m | 70 | N/A | N/A | N/A | N/A |
| **Ctrl#72** | f | 42 | N/A | N/A | N/A | N/A |
| **Ctrl#73** | f | 67 | N/A | N/A | N/A | N/A |

The table summarizes the characteristics of PD patients and controls (Ctrl) from Ulm University. N/K= not known; N/A=not applicable, DA=dopamin, MAO=monoaminooxidase, AChE= acetylcholinesterase, SSRI=selective serotonin re-uptake inhibitor
