# Supplementary material for: Inflammatory dysregulation of blood monocytes in Parkinson’s disease patients
Source: Acta Neuropathol. 2014 Oct 5;128(5):651–63. doi: 10.1007/s00401-014-1345-4 (PMC4201759; doi:10.1007/s00401-014-1345-4)
Supplement: Supplementary file 10 — Supplementary material 10 (DOCX 25 kb) [file 401_2014_1345_MOESM10_ESM.docx]

| **SUPPLEMENTARY TABLE 4. Characteristics of PD patients (cohort Fig. 4b)** | | | | | | |
| --- | --- | --- | --- | --- | --- | --- |
| **ID** | **gender** | **age** | **age of onset** | **disease duration [y]** | **medication** | **co-morbidities** |
| **PD#39** | f | 61 | 51 | 10 | L-dopa, DA agonist | adiposity, arterial hypertension, aortic insufficiency |
| **PD#40** | f | 61 | 51 | 10 | L-dopa, DA agonist | arterial hypertension, disc prolapse |
| **PD#41** | m | 71 | 57 | 14 | L-dopa | depression, sleep apnea |
| **PD#42** | f | 81 | 77 | 4 | L-dopa | dementia, adiposity, diabetes mellitus type II |
| **PD#43** | f | 66 | 56 | 10 | L-dopa, DA agonist, MAO inhibitor | no |
| **PD#11** | f | 76 | 66 | 10 | L-dopa | arterial hypertension |
| **PD#44** | f | 78 | 64 | 14 | L-dopa, DA agonist, MAO inhibitor | restless legs syndrome, degeneration of macula |
| **PD#45** | m | 63 | 52 | 11 | L-dopa | arterial hypertension |
| **PD#46** | m | 57 | 55 | 2 | L-dopa | depression, degenerative lumbar spine changes |
| **PD#47** | m | 64 | 56 | 8 | L-dopa, DA agonist | adiposity, arterial hypertension |
| **PD#3** | f | 78 | 63 | 15 | L-dopa, DA agonist | dementia |
| **PD#5** | m | 49 | 44 | 5 | DA agonist, MAO inhibitor | depression |
| **PD#4** | m | 69 | N/K | N/K | no | N/K |
| **PD#38** | f | 67 | 52 | 15 | L-dopa, DA agonist | plexus lesion, arterial hypertension |
| **PD#18** | m | 72 | 68 | 4 | L-dopa, DA agonist | hyperthyreoses, glaucoma |
| **PD#55** | m | 71 | N/K | N/K | L-dopa, DA agonist, MAO inhibitor | no |
| **PD#56** | m | 77 | N/K | N/K | L-dopa, DA agonist | no |
| **PD#57** | m | 78 | 70 | 8 | L-dopa, DA agonist, MAO inhibitor | arterial hypertension, prostate carcinoma (> 5 years ago) |
| **PD#58** | f | 68 | N/K | N/K | L-dopa, DA agonist | arterial hypertension, esophagitis, camptocormia |
| **PD#59** | m | 54 | 50 | 4 | MAO inhibitor | cognitive defects |
| **PD#60** | m | 73 | 68 | 5 | L-dopa, DA agonist | restless legs syndrome |
| **PD#61** | m | 43 | N/K | N/K | L-dopa, DA agonist, MAO inhibitor | no |
| **PD#62** | m | 85 | N/K | N/K | L-dopa, DA agonist | arterial hypertension, diabetes mellitus type II, glaucoma |
| **PD#63** | m | 74 | N/K | N/K | L-dopa, DA agonist, MAO inhibitor | camptocormia, dysphagia |
| **PD#64** | f | 70 | 63 | 7 | L-dopa, DA agonist | bulimia, mamma carcinoma (>5 years ago), depression |
| **PD#65** | m | 65 | N/K | N/K | L-dopa, DA agonist | depression, degenerative lumbar spine changes |
| **PD#39** | f | 61 | 51 | 10 | L-dopa, DA agonist | adiposity, arterial hypertension, aortic insufficiency |
| **PD#66** | f | 59 | 53 | 6 | DA agonist, NMDA agonist | sleeping problems |
| **PD#21** | f | 59 | 51 | 8 | L-dopa | adiposity |
| **PD#67** | m | 74 | 59 | 15 | L-dopa, MAO inhibitor | arterial hypertension, heart insuffiency, poly-neuropathy |
| **PD#68** | m | 70 | N/K | N/K | L-dopa | arterial hypertension, adiposity |
| **PD#69** | f | 78 | 63 | 15 | L-dopa, DA agonist, MAO inhibitor | no |
| **PD#70** | m | 81 | N/K | N/K | L-dopa | arterial hypertension, prostate hyperplasia |
| **PD#24** | f | 72 | 60 | 12 | L-dopa, DA agonist, AChE inhibitor, SSRI | mamma carcinoma (>5 years ago) |
| **ID** | **gender** | **age** | **age of onset** | **disease duration [y]** | **medication** | **co-morbidities** |
| **Ctrl#1** | m | 58 | N/A | N/A | N/A | N/A |
| **Ctrl#2** | m | 71 | N/A | N/A | N/A | N/A |
| **Ctrl#3** | m | 70 | N/A | N/A | N/A | N/A |
| **Ctrl#5** | m | 71 | N/A | N/A | N/A | N/A |
| **Ctrl#6** | m | 71 | N/A | N/A | N/A | N/A |
| **Ctrl#7** | f | 74 | N/A | N/A | N/A | N/A |
| **Ctrl#8** | m | 78 | N/A | N/A | N/A | N/A |
| **Ctrl#9** | f | 87 | N/A | N/A | N/A | N/A |
| **Ctrl#10** | f | 79 | N/A | N/A | N/A | N/A |
| **Ctrl#11** | m | 81 | N/A | N/A | N/A | N/A |
| **Ctrl#12** | f | 61 | N/A | N/A | N/A | N/A |
| **Ctrl#13** | m | 66 | N/A | N/A | N/A | N/A |
| **Ctrl#14** | m | 78 | N/A | N/A | N/A | N/A |
| **Ctrl#15** | m | 70 | N/A | N/A | N/A | N/A |
| **Ctrl#16** | m | 73 | N/A | N/A | N/A | N/A |
| **Ctrl#44** | m | 83 | N/A | N/A | N/A | N/A |
| **Ctrl#45** | m | 55 | N/A | N/A | N/A | N/A |
| **Ctrl#46** | f | 56 | N/A | N/A | N/A | N/A |
| **Ctrl#47** | f | 53 | N/A | N/A | N/A | N/A |
| **Ctrl#48** | f | 58 | N/A | N/A | N/A | N/A |
| **Ctrl#49** | f | 51 | N/A | N/A | N/A | N/A |
| **Ctrl#50** | f | 47 | N/A | N/A | N/A | N/A |
| **Ctrl#51** | m | 50 | N/A | N/A | N/A | N/A |
| **Ctrl#52** | f | 50 | N/A | N/A | N/A | N/A |
| **Ctrl#53** | m | 70 | N/A | N/A | N/A | N/A |
| **Ctrl#17** | f | 65 | N/A | N/A | N/A | N/A |
| **Ctrl#18** | m | 72 | N/A | N/A | N/A | N/A |
| **Ctrl#19** | m | 61 | N/A | N/A | N/A | N/A |
| **Ctrl#20** | f | 69 | N/A | N/A | N/A | N/A |
| **Ctrl#21** | f | 72 | N/A | N/A | N/A | N/A |
| **Ctrl#66** | m | 82 | N/A | N/A | N/A | N/A |
| **Ctrl#67** | f | 72 | N/A | N/A | N/A | N/A |
| **Ctrl#68** | f | 56 | N/A | N/A | N/A | N/A |
| **Ctrl#69** | m | 75 | N/A | N/A | N/A | N/A |
| **Ctrl#70** | m | 61 | N/A | N/A | N/A | N/A |
| **Ctrl#71** | m | 70 | N/A | N/A | N/A | N/A |
| **Ctrl#72** | f | 42 | N/A | N/A | N/A | N/A |
| **Ctrl#73** | f | 67 | N/A | N/A | N/A | N/A |
| **Ctrl#31** | f | 75 | N/A | N/A | N/A | N/A |
| **Ctrl#32** | f | 62 | N/A | N/A | N/A | N/A |
| **Ctrl#33** | m | 80 | N/A | N/A | N/A | N/A |
| **Ctrl#34** | m | 70 | N/A | N/A | N/A | N/A |
| **Ctrl#35** | m | 71 | N/A | N/A | N/A | N/A |
| **Ctrl#36** | f | 74 | N/A | N/A | N/A | N/A |
| **Ctrl#37** | m | 62 | N/A | N/A | N/A | N/A |
| **Ctrl#4** | f | 76 | N/A | N/A | N/A | N/A |
| **Ctrl#74** | m | 71 | N/A | N/A | N/A | N/A |
| **Ctrl#75** | m | 71 | N/A | N/A | N/A | N/A |
| **Ctrl#76** | f | 74 | N/A | N/A | N/A | N/A |
| **Ctrl#77** | m | 78 | N/A | N/A | N/A | N/A |
| **Ctrl#78** | f | 87 | N/A | N/A | N/A | N/A |
| **Ctrl#79** | f | 79 | N/A | N/A | N/A | N/A |
| **Ctrl#80** | m | 81 | N/A | N/A | N/A | N/A |
| **Ctrl#81** | f | 61 | N/A | N/A | N/A | N/A |

The table summarizes the characteristics of PD patients and controls (Ctrl) from Ulm University. N/K= not known; N/A=not applicable, DA=dopamin, MAO=monoaminooxidase, NMDA= N-Methyl-D-Aspartat
