# Supplementary material for: Inflammatory dysregulation of blood monocytes in Parkinson’s disease patients
Source: Acta Neuropathol. 2014 Oct 5;128(5):651–63. doi: 10.1007/s00401-014-1345-4 (PMC4201759; doi:10.1007/s00401-014-1345-4)
Supplement: Supplementary file 11 — Supplementary material 11 (DOCX 18 kb) [file 401_2014_1345_MOESM11_ESM.docx]

| **SUPPLEMENTARY TABLE 5. Characteristics of PD patients (cohort Fig. 5c, d and Supplemental Figure 6 a, b)** | | | | | | |
| --- | --- | --- | --- | --- | --- | --- |
| **ID** | **gender** | **age** | **age of onset** | **disease duration [y]** | **medication** | **co-morbidities** |
| **PD#48** | m | 61 | 53 | 8 | no | no |
| **PD#49** | m | 79 | N/K | N/K | no | no |
| **PD#50** | f | 78 | 75 | 3 | L-dopa | diabetes mellitus type II |
| **PD#51** | m | 85 | 78 | 7 | L-dopa, DA agonist | no |
| **PD#52** | f | 66 | 49 | 17 | L-dopa, MAO inhibitor | aortic insufficiency |
| **PD#53** | f | 70 |  | 70 | L-dopa, DA agonist | no |
| **PD#33** | f | 66 | 53 | 13 | L-dopa, DA agonist, NMDA agonist, MAO inhibitor, SSRI | coronary heart disease |
| **PD#39** | f | 61 | 51 | 10 | L-dopa, DA agonist | adiposity, arterial hypertension, aortic insufficiency |
| **PD#54** | f | 78 | 68 | 10 | DA agonist | dementia, degenerative lumbar spine changes |
| **PD#11** | f | 76 | 66 | 10 | L-dopa | arterial hypertension |
| **PD#36** | m | 74 | 68 | 6 | L-dopa, DA agonist, AChE inhibitor | depression, arterial hypertension |
| **PD#35** | f | 73 | 67 | 6 | L-dopa, DA agonist, MAO inhibitor | chronic kidney insufficiency, arterial hypertension, diabetes mellitus type II |
| **ID** | **gender** | **age** | **age of onset** | **disease duration [y]** | **medication** | **co-morbidities** |
| Ctrl#54 | m | 59 | N/K | N/K | N/K | N/K |
| Ctrl#55 | m | 68 | N/K | N/K | N/K | N/K |
| Ctrl#56 | f | 51 | N/K | N/K | N/K | N/K |
| Ctrl#39 | m | 59 | N/K | N/K | N/K | N/K |
| Ctrl#57 | m | 63 | N/K | N/K | N/K | N/K |
| Ctrl#58 | f | 70 | N/K | N/K | N/K | N/K |
| Ctrl#59 | f | 57 | N/K | N/K | N/K | N/K |
| Ctrl#60 | f | 58 | N/K | N/K | N/K | N/K |
| Ctrl#61 | f | 58 | N/K | N/K | N/K | N/K |
| Ctrl#62 | f | 64 | N/K | N/K | N/K | N/K |
| Ctrl#63 | f | 45 | N/K | N/K | N/K | N/K |
| Ctrl#64 | f | 69 | N/K | N/K | N/K | N/K |

The table summarizes the characteristics of PD patients and controls (Ctrl) from Ulm University. N/K= not known; N/A=not applicable, DA=dopamin, MAO=monoaminooxidase, NMDA= N-Methyl-D-Aspartat, AChE= acetylcholinesterase, SSRI=selective serotonin re-uptake inhibitor
