# Supplementary material for: Inflammatory dysregulation of blood monocytes in Parkinson’s disease patients
Source: Acta Neuropathol. 2014 Oct 5;128(5):651–63. doi: 10.1007/s00401-014-1345-4 (PMC4201759; doi:10.1007/s00401-014-1345-4)
Supplement: Supplementary file 12 — Supplementary material 12 (DOCX 12 kb) [file 401_2014_1345_MOESM12_ESM.docx]

**Supplementary table 6**

Top 10 dysregulated biological processes in Parkinson’s disease monocytes. GePS analysis.

| **Genomatix genome analyzer pathway system (GePS)** | |  |
| --- | --- | --- |
| **Biological Processes (GO)** | **p-value** | **genes** |
| **response to organic substance** | 5.12E-14 | 34/1623 |
| **response to chemical stimulus** | 1.37E-13 | 42/2646 |
| **response to molecule of bacterial origin** | 1.14E-12 | 14/199 |
| **response to stress** | 3.94E-12 | 41/2774 |
| **respopnse to wounding** | 4.23E-12 | 26/1056 |
| **response to lipopolysaccharide** | 9.20E-12 | 13/187 |
| **response to external stimulus** | 1.43E-12 | 27/1211 |
| **defense response** | 2.20E-11 | 25/1042 |
| **inflammatory response** | 4.54E-11 | 17/436 |
| **response to bacterium** | 4.54E-10 | 14/311 |
