# Supplementary material for: Inflammatory dysregulation of blood monocytes in Parkinson’s disease patients
Source: Acta Neuropathol. 2014 Oct 5;128(5):651–63. doi: 10.1007/s00401-014-1345-4 (PMC4201759; doi:10.1007/s00401-014-1345-4)
Supplement: Supplementary file 13 — Supplementary material 13 (DOCX 15 kb) [file 401_2014_1345_MOESM13_ESM.docx]

**Supplementary table 7**

Top 10 differentially regulated molecular functions in Parkinson’s disease monocytes. GePS analysis.

| **Genomatix genome analyzer pathway system (GePS))** |  |  |
| --- | --- | --- |
| **Molecular Functions (GO)** | **p-value** | **genes** |
| **chemokine activity** | 6.81E-08 | 6/47 |
| **chemokine receptor binding** | 1.78E-07 | 6/55 |
| **protein binding** | 2.43E-07 | 52/6730 |
| **MAP kinase tyrosine/serine/threonine phosphatase activity** | 2.53E-05 | 3/13 |
| **MAP kinase phosphatase activity** | 3.21E-05 | 3/14 |
| **CCR chemokine receptor binding** | 4.00E-05 | 3/15 |
| **cytokine receptor binding** | 4.35E-05 | 7/207 |
| **cytokine activity** | 4.49E-05 | 7/208 |
| **collagen binding** | 6.70E-05 | 4/48 |
| **protein tyrosine/threonine phosphatase activity** | 2.04E-04 | 2/5 |
