# Supplementary material for: Inflammatory dysregulation of blood monocytes in Parkinson’s disease patients
Source: Acta Neuropathol. 2014 Oct 5;128(5):651–63. doi: 10.1007/s00401-014-1345-4 (PMC4201759; doi:10.1007/s00401-014-1345-4)
Supplement: Supplementary file 14 — Supplementary material 14 (DOCX 12 kb) [file 401_2014_1345_MOESM14_ESM.docx]

| **Gene** | **Primer** | |
| --- | --- | --- |
| TBP | for | 5'-CCCATGACTCCCATGACC-3' |
|  | rev | 5'-TTTACAACCAAGATTCACTGTGG-3' |
| B2M | for | 5'-AGATGAGTATGCCTGCCGTG-3' |
|  | rev | 5'-GCGGCATCTTCAAACCTCCA-3' |
| JUN | for | 5'-ACGGCGGTAAAGACCAGAAG-3' |
|  | rev | 5'-CTCGCCCAAGTTCAACAACC-3' |
| FOSB | for | 5'-TTCTGACTGTCCCTGCCAAT-3' |
|  | rev | 5'-CGGGGTCAGATGCAAAATAC-3' |
| EGR1 | for | 5'-CTGACCGCAGAGTCTTTTCCTG-3' |
|  | rev | 5'-GCGGCCAGTATAGGTGATGG-3' |
| DUSP1 | for | 5'-GCCATTGACTTCATAGACTCCATC-3' |
|  | rev | 5'-AACTCAAAGGCCTCGTCCAG-3' |
| RHOB | for | 5'-GGTCCCCTGAGCATGCTTTT-3' |
|  | rev | 5'-GAGGGGAGTCGAACAGACAC-3' |
| FOS | for | 5'-GCGTTGTGAAGACCATGACAG-3' |
|  | rev | 5'-GGTCTGTCTCCGCTTGGAGT-3' |
| NFKBIZ | for | 5'-GGTCAGACGGCGAGTTCTTA-3' |
|  | rev | 5'-TCGAGATCTTCCTGTTAACCTTTGT-3' |

**Supplementary Table 8.** Primer sequences for primer used to quantify gene expression by qRT-PCR*. For: forward, rev: reverse.*
